# A tetraspanin gene regulating auxin response and affecting orchid perianth size and various plant developmental processes

**DOI:** 10.1002/pld3.157

**Published:** 2019-08-05

**Authors:** Wei‐Hao Chen, Wei‐Han Hsu, Hsing‐Fun Hsu, Chang‐Hsien Yang

**Affiliations:** ^1^ Institute of Biotechnology National Chung Hsing University Taichung Taiwan, ROC; ^2^ Advanced Plant Biotechnology Center National Chung Hsing University Taichung Taiwan, ROC

**Keywords:** *Arabidopsis thaliana*, auxin response, orchids, perianth, tetraspanin

## Abstract

The competition between L (lip) and SP (sepal/petal) complexes in P‐code model determines the identity of complex perianth patterns in orchids. Orchid tetraspanin gene *Auxin Activation Factor *(*AAF*) orthologs, whose expression strongly correlated with the expansion and size of the perianth after P code established, were identified. Virus‐induced gene silencing (VIGS) of *OAGL6‐2 *in L complex resulted in smaller lips and the down‐regulation of *Oncidium OnAAF*. VIGS of *PeMADS9* in L complex resulted in the enlarged lips and up‐regulation of *Phalaenopsis PaAAF*. Furthermore, the larger size of *Phalaenopsis* variety flowers was associated with higher *PaAAF* expression, larger and more cells in the perianth. Thus, a rule is established that whenever bigger perianth organs are made in orchids, higher *OnAAF*/*PaAAF* expression is observed after their identities are determined by P‐code complexes. Ectopic expression Arabidopsis *AtAAF* significantly increased the size of flower organs by promoting cell expansion in transgenic *Arabidopsis* due to the enhancement of the efficiency of the auxin response and the subsequent suppression of the jasmonic acid (JA) biosynthesis genes (*DAD1*/*OPR3)* and *BIGPETAL* gene during late flower development. In addition, auxin‐controlled phenotypes, such as indehiscent anthers, enhanced drought tolerance, and increased lateral root formation, were also observed in 35S::*AtAAF* plants. Furthermore, 35S::*AtAAF* root tips maintained gravitropism during auxin treatment. In contrast, the opposite phenotype was observed in palmitoylation‐deficient AtAAF mutants. Our data demonstrate an interaction between the tetraspanin AAF and auxin/JA that regulates the size of flower organs and impacts various developmental processes.

## 1 INTRODUCTION

Orchid flowers are typically of zygomorphic symmetry and contain nearly identically shaped sepals and petals. The most marvelous feature of orchid perianths is the conversion of the upper medial petal into a well‐differentiated labellum (lip), the evolution of which is thought to be to provide a platform for potential pollinators (Cozzolino & Widmer, 2005; Kocyan, Conti, Qiu, & Endress, 2004; Rudall & Bateman, 2002). We have found a conserved principle known as the perianth (P) code, which states that the determination of lips and sepals/petals in orchids is controlled by L (lip) and SP (sepal/petal) complexes, respectively (Hsu et al., 2015). The higher‐order heterotetrameric L (lip) complex is exclusively required for lip determination, while the SP (sepal/petal) complex specifies sepal/petal formation (Hsu et al., 2015). How exactly orchid perianth formation and characteristics, such as morphological features and size, are regulated after P‐code complexes are established remains obscure. For example, lips are much larger than sepals/petals in *Oncidium* orchids. By contrast, lips are much smaller than sepals/petals in *Phalaenopsis* orchids. It is therefore a reasonable assumption that these two orchid genera have opposing mechanisms for regulating perianth size in the lips and sepals/petals.

*TETRASPANIN* genes have been identified in multicellular eukaryotes (Huang et al., 2005; Lambou et al., 2008; Wang et al., 2015) but were absent in yeast (Garcia‐España et al., 2008). The structures of tetraspanin family proteins were evolutionary conserved (Zuidscherwoude et al., 2015) and contained five distinct regions, including a large extracellular domain, a small extracellular domain, transmembrane domains, palmitoylation sites, and cytoplasmic domains (Stipp, Kolesnikova, & Hemler, 2003). In mammalian cells, tetraspanins form a tetraspanin‐enriched microdomain (TEM), known as a tetraspanin web, by interacting with one another, specific lipids and other transmembrane proteins, including integrins and other adhesion receptors (Charrin, Jouannet, Boucheix, & Rubinstein, 2014; Hemler, 2005; Reimann, Kost, & Dettmer, 2017; Zuidscherwoude et al., 2015). The organization of the integrin–tetraspanin microdomain and modulation of adhesion‐dependent signaling were mediated by a posttranslation modification of tetraspanins via palmitoylation (Berditchevski, Odintsova, Sawada, & Gilbert, 2002). The mutation of juxtamembrane cysteines, the palmitoylation site of a human tetraspanin CD81, reduced the ability of CD81 to interact with other proteins (Delandre, Penabaz, Passarelli, Chapes, & Clem, 2009). Additionally, the palmitoylation‐deficient human tetraspanin CD82 lost the function of inhibition of cancer cell migration and invasion (Zhou, Liu, Reddivari, & Zhang, 2004).

In plants, tetraspanins have been reported to have diverse functions during growth and development (Reimann et al., 2017). In rice and *Arabidopsis*, 15 and 17 *TETRASPANIN* genes were identified, respectively (Boavida, Qin, Broz, Becker, & McCormick, 2013; Cnops et al., 2006). Arabidopsis *TETRASPANIN1(TET1)*/*TORNADO2*/*EKEKO* has a function in leaf morphogenesis and root patterning (Cnops et al., 2006; Lieber, Lora, Schrempp, Lenhard, & Laux, 2011; Olmos, Reiss, & Dekker, 2003). *TET1* also involved in modulating auxin homeostasis and the transition from floral meristem termination to gynoecium development (Yamaguchi, Huang, Xu, Tanoi, & Ito, 2017). *TET5* and *TET6* have redundant functions in restricting cell proliferation during root and leaf growth (Wang et al., 2015). *TET13* has a function in the primary root, affecting apical meristem size and root length, and in lateral root initiation (Wang et al., 2015). However, the functions of most plant *TETRASPANIN* genes still remain to be investigated (Reimann et al., 2017).

It is well known that phytohormone auxin plays a critical role in plant growth, including photo‐ and gravitropism, root formation, embryo development, petal development, and anther dehiscence (Cecchetti et al., 2013; Lampugnani, Kilinc, & Smyth, 2012; Lavenus et al., 2013; Mironova, Teale, Shahriari, Dawson, & Palme, 2017; Moller et al., 2017; Overvoorde, Fukaki, & Beeckman, 2010; Péret et al., 2013; Sauret‐Gueto, Schiessl, Bangham, Sablowski, & Coen, 2013; Teale, Paponov, & Palme, 2006). The gradients and distributions of auxin determine organ initiation, tissue patterning, and differential growth during tropic responses (Armengot, Marques‐Bueno, & Jaillais, 2016; Finet & Jaillais, 2012; Zhao, 2010). During root formation, auxin is synthesized in the root apex (Petersson et al., 2009), then effluxed by PIN‐FORMED proteins (PIN), and influxed by AUX1/LIKE AUX1 proteins (AUX1/LAX) that generate a longitudinal gradient of auxin in roots (Armengot et al., 2016). When roots are under 90‐degree gravitropic stimulation, auxin redistributes to the lower side of the roots and inhibits the elongation of epidermal cells, causing root bending (Sato, Hijazi, Bennett, Vissenberg, & Swarup, 2015). In addition, lateral root primordia are triggered by gravitropic stimulus and develop in eight stages (Péret et al., 2012).

Auxin has been thought to regulate petal development since the disrupted expression of an auxin‐inducible indicator DR5 were observed in *petal loss* (*PTL*) mutant plants (Lampugnani, Kilinc, & Smyth, 2013). The sizes of petals and the lengths of stamens were significantly reduced in *Arabidopsis auxin response factor 6* (*arf6*) and *arf8* double‐mutant plants (Nagpal et al., 2005). Auxin may regulate petal development and anther dehiscence through the regulation of jasmonate (JA) activity, since it has been shown that exogenous auxin inhibits the expressions of the JA biosynthesis gene *DEFECTIVE IN ANTHER DEHISCENCE1* (*DAD1*) and *12‐oxophytodienoate reductase* (*OPR3*) and results in anther indehiscence (Cecchetti et al., 2013). Interestingly, petal size is increased in *opr3* mutants due to larger cell sizes at developmental stage 14 instead of stage 9, indicating that JA controls petal size by suppressing cell expansion at late stages (Brioudes et al., 2009). This assumption was supported by the fact that JA regulates an alternative splicing event in the ubiquitously expressed gene *BIGPETAL* (*BPE*) to translate a basic helix‐loop‐helix (bHLH) transcription factor BPEp that inhibits cell expansion in late development stages of petal growth (Brioudes et al., 2009; Varaud et al., 2011).

Auxin is also involved in response to abiotic stresses such as drought, salt, and cold. Exogenous auxin enhanced tolerance to drought (Shi et al., 2014) and rescued the salt hypersensitivity phenotype of plants overexpressing *TTG2*/*WRKY44* (Li et al., 2015). Exogenous or enhanced production of endogenous auxin improved salt tolerance in sorghum and mung bean (Azooz, Shaddad, & Abdel‐Latef, 2004; Zahir, Shah, Naveed, & Akhter, 2010). Higher drought tolerance was observed in auxin‐overexpressing mutants *yuc6*‐*1D* and *yuc7*‐*1D*. Furthermore, *yuc6*‐*1D* mutant plants also showed delayed senescence (Kim et al., 2011; Lee et al., 2012).

In this study, we identified the tetraspanin gene *Auxin Activation Factor *(*AAF*) from *Oncidium* and *Phalaenopsis* orchids and demonstrated that the expression of orchid *AAF* orthologs is associated with the regulation of the perianth size after its identity is determined by the P‐code complexes. Further analysis of an *Arabidopsis AAF* gene reveals that *AAF* controls not only flower organ size but also various developmental processes such as anther dehiscence, drought tolerance, and lateral root formation by enhancing the efficiency of the auxin response in plants. Our results suggest a novel interaction between the tetraspanin gene *AAF* and auxin in regulating plant growth and development.

## 2 MATERIALS AND METHODS

### 2.1 Plant materials and growth conditions

Seeds for *Arabidopsis* were sterilized and placed on agar plates containing Murashige and Skoog medium (Murashige & Skoog, 1962) at 4°C for 2 days. The seedlings were then grown in growth chambers under long‐day conditions (16‐hr light/8‐hr dark) at 22°C for 10 days before being transplanted to soil. The light intensity of the growth chambers was 150 μE m^−2^ s^−1^. Species, cultivars, and peloric mutants of orchids used in this study, including the *Oncidium* (*O.* Lemon Heart and the associated peloric mutants *O.* Lemon Heart Trilips) and moth orchids (*Phalaenopsis* Sogo Yukidian “V3,” *P.* Red Bell, *P.* Gold Diamond, and the associated peloric mutants *P.* Big‐Lip), were maintained in the greenhouse of National Chung‐Hsing University, Taichung, Taiwan.

### 2.2 Cloning of orchid *AAF* cDNAs

The transcriptomic RNA‐Seq for *Oncidium* (*O.* Lemon Heart) and *Phalaenopsis* (*P.* Sogo Yukidian “V3”) floral organs (lip and sepal/petal) at floral bud stage (6 mm for *Oncidium* and 15 mm for *Phalaenopsis*) was performed and data analyzed. An *Oncidium Auxin Activation Factor *(*OnAAF*) gene which expressed specifically higher in lip than in sepal/petal of *Oncidium* was identified. *PaAAF* which showed highest sequence identity/similarity to *OnAAF* and expressed specifically higher in petal than in lip of *Phalaenopsis* was also identified. The cDNA contained the 3′‐end of *OnAAF* was obtained by 3′‐RACE using the BD SMART RACE cDNA Amplification Kit (Clontech Laboratories) following the 5′ gene‐specific primer OnAAF‐1. The full‐length cDNA of *OnAAF* was amplified by PCR using 5′ primer, OnAAF‐1, and the 3′ primer, OnAAF‐2. The full‐length cDNA of *Phalaenopsis PaAAF* was amplified by PCR using 5′ primer, PaAAF‐1, and the 3′ primer, PaAAF‐2. Sequences for the primers are listed in Table S1.

### 2.3 Cloning of *Arabidopsis AtAAF* cDNA

*Arabidopsis Auxin Activation Factor *(*AtAAF*) (At4g30430), contains two exons separated by an intron, was identified on chromosome 4. cDNA containing an open reading frame of *AtAAF* was amplified by PCR using the 5' primer, MSIF‐3, and the 3' primer, MSIF‐4. The amplified fragment containing the cDNA of *AtAAF* gene was cloned into the linker region in binary vector pEpyon‐12K (CHY Lab) under the control of cauliflower mosaic virus (CaMV) 35S promoter (35S::*AtAAF*) and used for further plant transformation. Sequences for the primers are listed in Table S1.

### 2.4 Construction of 35S::*AtAAF^palm^*

To generate the *AtAAF^palm^* fragment, two fragments were amplified for mega PCR using cDNA of *AtAAF* as the template with primers, 5′MSIF‐3‐XbaI, 3′MSIF‐18‐C65S/C66S, 5′MSIF‐17‐C65S/C66S, and 3′MSIF‐20. The 3′MSIF‐18‐C65S/C66S primer (5′‐GAAGCCACGTCACTCTGGAAGAAGATC‐3′) contained two 1 bp substitution from GCAACA to GGAAGA and 5′MSIF‐17‐C65S/C66S primer (5′‐GATCTTCTTCCAGAGTGACGTGGCTTC‐3′) contained two 1 bp substitution from TGTTGC to TCTTCC that convert Cys65/Cys66 of the AtAAF protein to Ser65/Ser66. Another two fragments were amplified with primers 5′‐MSIF‐19, 3′‐MSIF‐21‐C252S/C253S, 5′‐MSIF‐22‐C252S/C253S, and 3′‐MSIF‐4‐KpnI. The 3′‐MSIF‐21‐C252S/C253S primer (5′‐GAAAGCGGAAGATCCCATAGCGTAGAC‐3′) contained two 1 bp substitution from GCAACA to GGAAGA and 5′‐MSIF‐22‐ C252S/C253S primer (5′‐GTCTACGCTATGGGATCTTCCGCTTTC‐3′) contained two 1 bp substitution from TGTTGC to TCTTCC that convert Cys252/Cys253 of the AtAAF protein to Ser252/Ser253. There is an endogenous *Xho*I recognition site in the first fragment. The XbaI‐XhoI and XhoI‐KpnI PCR fragments were inserted in the binary vector pEpyon‐12K (CHY Lab), by endogenetic *Xho*I (5′‐CTCGAG‐3′) digestion site ligation. A multiple point mutation fragment was generated that contained two bp substitution from TGTTGC to TCTTCC that converts Cys65, Cys66, Cys252, and Cys253 of the AtAAF protein to Ser65, Ser66, Ser252, and Ser253. The product contained the generated *Xba*I recognition site (5′‐TCTAGA‐3′) and *Kpn*I recognition site (5′‐GGTACC‐3′) to facilitate the cloning of *AtAAF* cDNA. Sequences for the primers are listed in Table S1.

### 2.5 AtAAF::*GUS* fusion construct

For the AtAAF::*GUS* construct, the *AtAAF* promoter (2.0 kb) was obtained by PCR amplification from the genomic DNA using the pMSIF‐1 and pMSIF‐2 primers and then cloned into pGEM‐T easy vector (Promega). The full‐length promoter for *AtAAF* (2.0 kb) was then subcloned into the linker region before the β‐Glucuronidase (GUS) coding region in binary vector pEpyon‐01K (CHY Lab). The primers contained the generated *Pst*I (5′‐CTGCAG‐3′) recognition site and *Sal*I (5′‐GTCGAC‐3′) recognition site to facilitate the cloning of the promoter. Sequences for the primers are listed in Table S1.

### 2.6 Construction of *AtAAF+GFP* construct

*AtAAF* cDNAs were subcloned into the multiple cloning site of binary vector pEpyon‐12K (CHY Lab) upstream of the mGFP5 sequence and under the control of the CaMV 35S promoter. The fragments contained the generated *Xba*I and *Kpn*I recognition site to facilitate the cloning of the *AtAAF*. This construct was used for plant transformation. The sequences for the primers are listed in Table S1.

### 2.7 Real‐time PCR analysis

For real‐time quantitative RT‐PCR, the reaction was performed on an MJ Opticon system (MJ Research) using SYBR Green Real‐Time PCR Master Mix (TOYOBO Co., LTD.). The amplification conditions were 95°C for 10 min, followed by 40 cycles of amplification (95°C for 15 s, 58°C for 15 s, and 72°C for 30 s, followed by plate reading) and melting (50–95°C with plate readings every 1°C). The sequences for the primers that were used for the real‐time quantitative RT‐PCR for *OnAAF, OAGL6‐2*, *PaAAF, PeMADS9, AtAAF*, *EDF1*, *EDF2*, *ERF1*, *SAG12*, *GFP*, *DAD1*, *OPR3*, *MYB26*, *NST1, NST2,* and *BPEp* are listed in Table S1. The Arabidopsis housekeeping gene *UBQ10* was used as a normalization control with the following primers: RT‐UBQ10‐1 and RT‐UBQ10‐2. The transcript levels for orchid genes were determined using three replicates and were normalized using reference genes *ACTIN* (primers: RT‐PACT4‐1 and RT‐PACT4‐F) for *Phalaenopsis* (Hsu et al., 2015) and *α‐tubulin* (primers: RT‐OTUB‐1 and RT‐OTUB‐2) for *Oncidium* (Chang et al., 2010) as described previously (Hsu et al., 2015). The data were analyzed using CFX Manager™ software (version 3.1; Bio‐Rad) according to the manufacturer's instructions. The “delta–delta method” formula 2^−[△CP sample‐△CP control]^, where represents perfect PCR efficiency, was used to calculate the relative expression of the genes. To calculate the statistical significance, unpaired *t* tests were used.

### 2.8 Plant transformation and transgenic plant analysis

Constructs made in this study were introduced into *Agrobacterium tumefaciens* strain GV3101 and transformed into *Arabidopsis* plants using the floral dip method as described elsewhere (Clough & Bent, 1998). Transformants that survived in the medium containing kanamycin (50 μg/ml) were further verified by RT‐PCR analysis.

### 2.9 Stress treatments

For drought treatment, 1‐week‐old Arabidopsis seedling was removed from the MS medium and exposed to a stream of air for various times. Subsequently, seedlings were weighted, the pictures of seedlings were taken and the RNA of seedlings was extracted for gene expression analysis. For salt stress treatment, 1‐week‐old seedlings were removed from the MS medium and NaCl were added to the liquid MS medium to a final concentration of 150 mM. Seedlings were dipped for various times, and the RNA of seedlings was extracted for gene expression analysis. Plants were grown in MS medium plate with 150 mM NaCl for survival rate calculation.

### 2.10 Alexander's staining

For pollen analysis, the pollen grains were mounted with Alexander's stain as previously described (Alexander, 1969).

### 2.11 Cryo‐scanning electron microscopy

Cryo‐scanning electron microscopy (SEM) was performed according to the method as previously described (Hsu et al., 2015).

### 2.12 Confocal laser scanning microscopy

The flower tissues were imaged using an Olympus FV1000 confocal microscope as described previously (Chang et al., 2010; Peng et al., 2013). The plant cell walls were stained with 40 μg/ml propidium iodide (PI; Molecular Probes). PI was excited by a 543‐nm He/Ne laser line, and the emission was collected at 555–655 nm.

### 2.13 Lignin staining

For lignin analysis, fresh anthers were stained with 0.01% auramine O (Pesquet et al., 2005) and observed with a confocal microscope (Olympus FV1000). The lignified cells were observed under 488 nm excitation/510–560 nm emission. Cellulose of cell walls was stained with 0.07% calcofluor white and observed under 370 nm excitation/420 nm emission.

### 2.14 Application of jasmonate

All opened flowers (after stage 14) were removed from the inflorescence, and the remaining flower bud clusters were dipped into 50 µM (±) Jasmonate (Sigma) dissolved in 0.05% aqueous Tween 20.

### 2.15 Histochemical GUS assay

Histochemical staining was performed under the standard method described previously (Jefferson, Kavanagh, & Bevan, 1987).

### 2.16 Measurement of the concentration of the IAA

Flower buds of Arabidopsis were ground under liquid nitrate then resuspended in 90 μl phosphate‐buffered saline (PBS) buffer for total IAA extraction. The IAA quantification was performed by enzyme‐linked immunosorbent assay (ELISA) kit (LYBDBio).

### 2.17 Biotin switch assay of S‐acylation

The biotin switch assay was performed as previously described (Hemsley, Taylor, & Grierson, 2008) with minor modification. Briefly, about 0.8 mg total proteins were solubilized and incubated with 25 mM *N*‐ethylmaleimide (Thermo Fisher Scientific) to block free sulfhydryls. Free *N*‐ethylmaleimide was removed by concentration tube (Millipore) then divide into two equal aliquots. One aliquot was incubated with 1 M hydroxylamine (NH_2_OH) (Thermo Fisher Scientific) to cleave thioester bonds and with 1 mM EZ‐link™ biotin‐HPDP (Thermo Fisher Scientific) to label liberated sulfhydryls. Hydroxylamine was replaced by Tris‐HCl buffer in the remaining aliquot as a control. Free reagent was removed as mentioned above, and 12 μl of the solution was removed as a loading control. Biotinylated proteins were then purified with 15 μl NeutrAvidin‐agarose (Thermo Fisher Scientific) and analyzed by Western blotting using GFP‐specific antibodies.

### 2.18 Virus‐induced gene silencing experiment

The virus‐induced gene silencing (VIGS) experiments in orchids were performed as described previously (Hsu et al., 2015). DNA fragments for *OAGL6‐2* and *PeMADS9* (*OAGL6‐2* homolog) for insertion into the VIGS vector pCymMV‐Gateway (Lu et al., 2012) were obtained by PCR amplification using the following primers: OAGL6‐2 (in the C domain, 160 bp), O1‐gateway‐F, 5′‐GGGGACAAGTTTGTACAAAAAAGCAGGCTAGCAAATGGTGGGTCATC‐3′; O1‐gateway‐R, 5′‐GGGGACCACTTTGTACAAGAAAGCTGGGTAAATGGTTGCTTCAGAAG‐3′. PeMADS9 (in the C domain, 150 bp), PeM9‐VIGS‐F, 5′‐GGGGACAAGTTTGTACAAAAAAGCAGGCTCAGGTGATATTAACAAGCAGCTTAAACA‐3′; PeM9‐VIGS‐R, 5′‐GGGGACCACTTTGTACAAGAAAGCTGGGTCTTTGAAGAGTGGGTTCTGTATCCATG‐3′. The underlined sequences are *att*B sites for in vitro recombination with *att*P sites in the VIGS vector pCymMV‐Gateway to generate recombinant clones using Gateway^®^ BP Clonase II Enzyme Mix (Invitrogen™, Life Technologies). pCymMV‐Gateway‐OAGL6‐2, pCymMV‐Gateway‐PeMADS9, and the empty pCymMV‐Gateway as a control were transformed into *Agrobacterium tumefaciens* EHA105 for further inoculation. For *Oncidium* Lemon Heart leaf infiltration, suspensions were injected into the third leaf (L3) just below the site where the inflorescence emerged. For *Phalaenopsis* Sogo Yukidian “V3” leaf infiltration, suspensions were injected into the leaf just above the site where the inflorescence emerged. For every infiltration, at least three plants were inoculated with each pCymMV‐Gateway construct. Flower samples were collected and analyzed at 45 DPI (days postinoculation), when the buds at the end of the *Oncidium* inflorescences or the last bud of the *Phalaenopsis* inflorescences bloomed.

## 3 RESULTS

### 3.1 The expression of *Oncidium OnAAF* is positively correlated with the growth of lip and is down‐regulated in *OAGL6‐2* VIGS small lips

**Figure 1:**
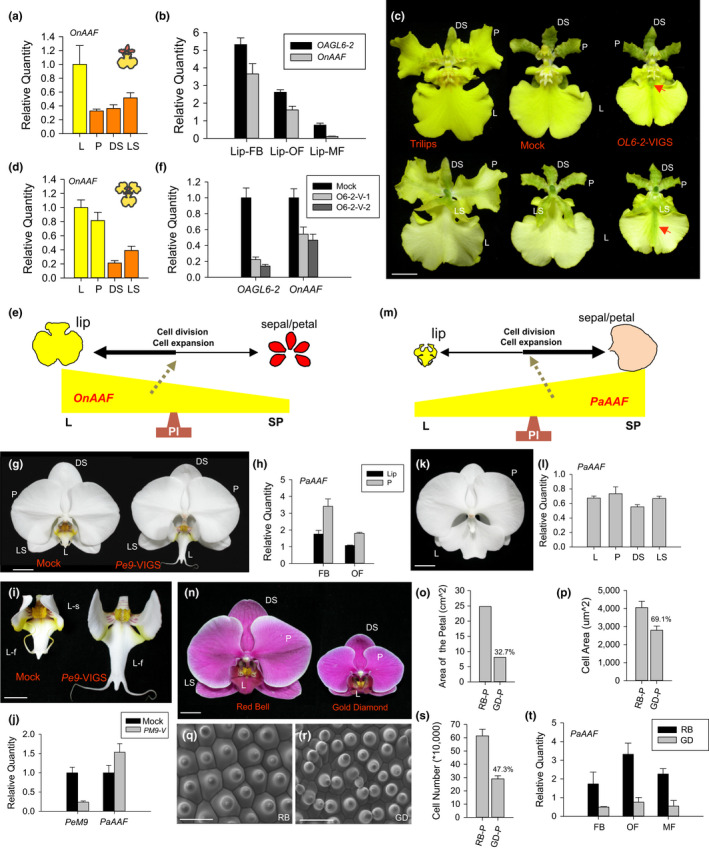
Functional analysis of *OnAAF* and *PaAAF* in *Oncidium* and *Phalaenopsis* orchids. (a) Total RNA samples isolated from the lips (L), petals (P), dorsal sepals (DS), and lateral sepals (LS) of mature *Oncidium* flowers were used as templates to detect the expression of *OnAAF* by quantitative real‐time PCR. (b) Detection of *OAGL6‐2* and *OnAAF* expression in the lips of *Oncidium* floral bud (FB), open flower (OF), and mature flower (MF). (c) The flowers of *Oncidium* Lemon Heart peloric mutant (Trilips) with two petals transformed into lips (left), the *OAGL6‐2* VIGS flower (right) containing green sepal/petal‐like sectors in the lips (arrow), which are smaller than those in the wild‐type control (Mock, middle). Bar = 10 mm. Top and bottom rows indicate the adaxial and abaxial side of the flower, respectively. (d) Detection of *OnAAF* expression in L, P, DS, and LS of mature flowers of *Oncidium* Lemon Heart peloric mutant (Trilips). (e) *OnAAF* expression was higher and promoted more cell division/expansion in lips than sepals/petals, resulting in the production of larger lips than sepals/petals in *Oncidium* orchids. (f) Detection of *OAGL6‐2* and *OnAAF* expression in the lips of *OAGL6‐2* VIGS (O6‐2‐V‐1 and 2) and wild‐type control (Mock) flowers of *Oncidium* Lemon Heart. (g) The flower of *PeMADS9* (*OAGL6‐2* ortholog) VIGS *Phalaenopsis *Sogo Yukidian “V3” (Pe9‐VIGS, right) contains lips, which are larger and more spread out than in the wild‐type control flower (Mock, left) Bar = 20 mm. (h) Detection of *PaAAF* expression in lips (Lip) and P of *Phalaenopsis *Sogo Yukidian “V3” FB and OF. (i) The front lobe (L–f) and side lobe (L‐s) of the lips from (g)*.* Bar = 10 mm. (j) Detection of *PeMADS9* (*PeM9*) and *PaAAF* expression in lips of *PeMADS9* VIGS (PM9‐V) and wild‐type control (Mock) flowers of *Phalaenopsis *Sogo Yukidian “V3.” (k) The flowers of a *Phalaenopsis* peloric mutant (Big‐Lip) have much larger sepal/petal‐like L. (l) Detection of *PaAAF* expression in L, P, DS, and LS from the Big‐Lip mutant flower in (k). (m) Higher *PaAAF* expression promoted greater cell division/expansion in sepals/petals than in lips, resulting in the production of larger petals than lips in *Phalaenopsis* orchids. (n) Two varieties of *Phalaenopsis* orchids with relatively large (*P.* Red Bell) and small (*P.* Gold Diamond) mature flower sizes. Bar = 20 mm. (o) The size comparison of the P of *P.* Red Bell (RB) and *P.* Gold Diamond (GD) from (n). (p) The comparison of the epidermal cell size in the petals of *P.* Red Bell (RB) and *P.* Gold Diamond (GD) from (n). (q, r) SEM of the epidermal cells in the petals of *P.* Red Bell (q) and *P.* Gold Diamond (r) from (n). Bar = 100 μm. (s) The comparison of the total cell number in the petals of *P.* Red Bell (RB) and *P.* Gold Diamond (GD) from (n). (t) Detection of *PaAAF* expression in *P.* Red Bell (RB) and *P.* Gold Diamond (GD) FB, OF, and MF

To explore how orchid perianth formation and characteristics, such as morphological features and size, are regulated after P‐code complexes are established, NGS analysis was used to identify differentially expressed genes in the lips or sepals/petals of *Oncidium* orchids. An *Oncidium* TET protein (Figures S1 and S2) was selected for further analysis because it was (a) predominantly expressed in the lips rather than in the sepals/petals (Figure 1a) and (b) was positively correlated with the expression of the L complex gene *OAGL6‐2* during different stages of lip development (Figure 1b). We renamed the protein "Auxin Activation Factor" (AAF) for data presented in this study.

The *Oncidium Auxin Activation Factor* (*OnAAF*) is a member of the tetraspanin family in the TET7/8/9 group (Figures S1 and S2) encoding a protein of 270 amino acids (Figure S1) which showed the 57% identity and 75% similarity to its Arabidopsis TETRASPANIN orthologue AtAAF (At4g30430) (originally known as TET9) (Figures S1 and S2). The OnAAF protein contained four transmembrane domains, two extracellular loops, and cytoplasmic N and C terminals (Figure S1). When the sequence of the OnAAF protein was further analyzed, four palmitoylation sites were predicted by CSS‐Palm (Zhou, Xue, Yao, & Xu, 2006) (Figure S1).

In the *Oncidium* Trilips mutant (Figure 1c), *OnAAF* was clearly up‐regulated in the enlarged lip‐like petals (Figure 1d), indicating that *OnAAF* likely promoted lip expansion after the P code was established (Figure 1e). To further test this hypothesis, we attempted to suppress the activity of the L complex by VIGS of *OAGL6‐2 *in the lips of *Oncidium* Lemon Heart. VIGS of *OAGL6‐*2 reduced the size of the lip in flowers (Figure 1c), resulting in a smaller sepal/petal‐like lip structure (Figure 1c) that was correlated with the down‐regulation of *OAGL6‐2* expression (Figure 1f). Interestingly, expression of *OnAAF* decreased significantly in the sepal/petal‐like lips of flowers with *OAGL6‐2* VIGS (Figure 1f).

Furthermore, the expression of *OnAAF* in lips was found to be expressed higher during early developmental stage (10 mm bud), gradually decreased in open flower (15 mm) and decreased significantly in mature flower (Figure 1b). The size of the lips was much smaller in 10 mm bud than in mature flower (Figure S3A,B,E) (Chang et al., 2010). The cell size in lips was also smaller in 10 mm bud than in mature flower (Figure S3C,D,F) (Chang et al., 2010). However, the total cell number was similar in these two stages of lips (Figure S3G). This result indicated that cell expansion rather than cell division was occurred in lip from early stage of flower buds to later stage of mature flower. Thus, the high level of the *OnAAF* expression in early lip development revealed that *OnAAF* expression is associated with the regulation of the lip size primarily due to the control of cell division rather than cell expansion during lip development.

### 3.2 The expression of *Phalaenopsis PaAAF* is positively correlated to the growth of perianth and is up‐regulated in *PeMADS9 *(*OAGL6‐2* ortholog) VIGS‐enlarged lips

To investigate the function of orchid *AAF*, an *OnAAF* ortholog *PaAAF* which showed highest sequence identity/similarity to *OnAAF*, was identified through the analysis of NGS data and characterized in *Phalaenopsis* Sogo Yukidian “V3” (Figure 1g). The PaAAF protein encodes 271 amino acids and shows 86% identity and 95% similarity to OnAAF (Figures S1 and S2).

Compared with *Oncidium* orchids, *Phalaenopsis* orchids have significantly smaller lips relative to their sepals/petals (Figure 1g). Not surprisingly, the expression of *PaAAF* was lower in the lips than in the petals during the same developmental stages (Figure 1h). These results suggest that *PaAAF* expression is also associated with the promotion of perianth organ growth in *Phalaenopsis* orchids. In petals, the expression of *PaAAF* was found to be expressed higher during early developmental stage (15 mm bud) and decreased in open flower (Figure 1h). The size of the petals was much smaller in 15 mm bud than in open flower (Figure S4A,B,E). The cell size in petals was much smaller in 15 mm bud than in open flower (Figure S4C,D,F). The total cell number was however similar in these two stages of petals (Figure S4G). This result indicated that cell expansion rather than cell division was correlated with the increasing size of petal from early stage to later stage of flower development. Thus, the high level of the *PaAAF* expression in early petal development revealed that *PaAAF* expression is associated with the regulation of the petal size primarily due to the control of cell division rather than cell expansion during lip development.

A VIGS strategy was further used to suppress *PeMADS9* (*OAGL6‐2* ortholog) expression in *Phalaenopsis* Sogo Yukidian “V3.” The *PeMADS9‐*silenced lips were much more spread out and expanded to a larger size compared with the sepals/petals (Figure 1g,i). The enlarged sepal/petal‐like lip structure that resulted from *PeMADS9* VIGS was associated with significant down‐regulation of *PeMADS9* and up‐regulation of *PaAAF* (Figure 1j). Analysis of the *Phalaenopsis* big‐lip mutants in which the lip was converted into a petal‐like structure (Figure 1k) revealed that *PaAAF* was clearly up‐regulated in these petal‐like lips to a level similar to that in the petals/sepals (Figure 1l). This result indicates that the expression level of *PaAAF* is correlated with the expansion and size of the perianth (Figure 1m). Greater *PaAAF* expression is associated with the production of larger perianth organs. This observation was further supported by analyzing two varieties of *Phalaenopsis* orchids that significantly differed in the size of their mature flowers but were otherwise morphologically similar (Figure 1n). This analysis indicated that the larger size of *Phalaenopsis* Red Bell flowers (Figure 1o) was associated with larger cells (Figure 1p,q,r), more cells (Figure 1s) and higher *PaAAF* expression (Figure 1t) in the perianth as compared to *Phalaenopsis* Gold Diamond flowers. These results suggest that *AAF* orthologs are regulators of perianth size in orchids.

### 3.3 *AtAAF* expression is high in flower buds and AtAAF protein is localized at the plasma membrane and can be palmitoylated

To further validate the function of the *AAF* ortholog, *Arabidopsis AtAAF/TET9* (At4g30430) was characterized extensively in this study. Based on *Arabidopsis* eFP browser data (Schmid et al., 2005; Winter et al., 2007) (http://www.bar.utoronto.ca/efp/cgi-bin/efpWeb.cgi), *AtAAF* (will be referred throughout this work) showed the most similar expression pattern in flower (higher in early than in late flower development) to *OnAAF/PaAAF* than other Arabidopsis TETRASPANIN genes. *AtAAF* contains two exons separated by a 425 base‐pair intron and encodes a protein of 272 amino acids which showed 62%/67% identity and 80%/83% similarity to the two most closely related TETRASPANIN proteins, At2g23810 (TET8) and At4g28050 (TET7), respectively (Figures S1 and S2).

**Figure 2:**
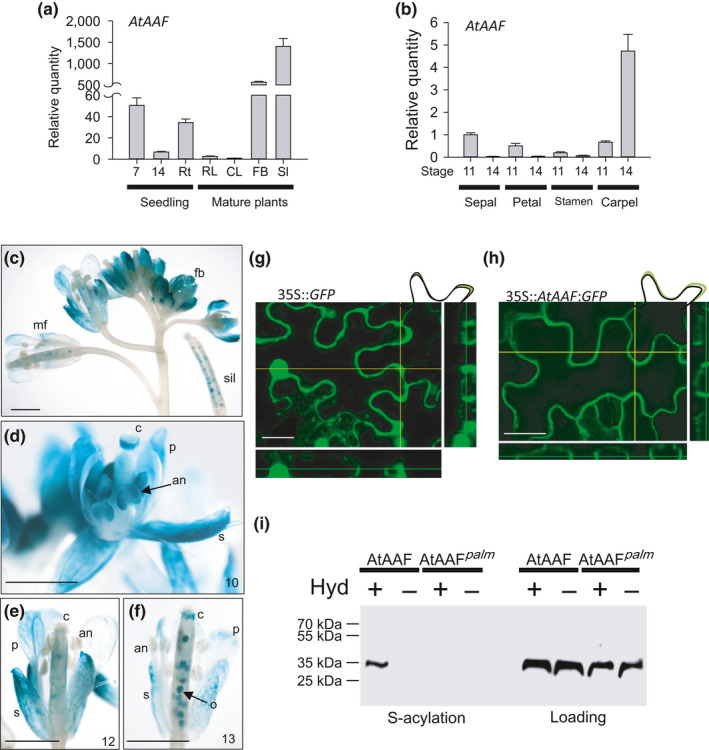
Analysis of the gene expression, protein localization, and palmitoylation for *AtAAF* in *Arabidopsis*. (a) The detection of *AtAAF* expression at 7 days after germination (DAG), 14 DAG, roots (Rt), rosette leaves (RL), cauline leaves (CL), floral buds (FB), and siliques (Sl). (b) The detection of *AtAAF* expression in sepal, petal, stamen, and carpel of stages 11 and 14 Arabidopsis flowers. (c) GUS staining pattern in floral buds (fb), mature flower (mf), and silique (sil) from an AtAAF::*GUS* transgenic plant. Bar = 1 mm. (d–f) GUS was detected in sepals (s), petals (p), and anther (an) of stage 10 (d) AtAAF::*GUS* flowers. GUS activity decreased in s and p and was absent in the anther after stage 12 (e, f). GUS was detected in ovules (o) after pollination (f). c, carpel. Bar = 1 mm. (g, h) Transient expression of 35S::*GFP* and 35S::*AtAAF*+*GFP* in tobacco cells. AtAAF+GFP fusion protein accumulated at the plasma membrane (h) whereas GFP protein accumulated in the cytosol (g). Bar = 30 μm. (i) Assaying palmitoylation of AtAAF and dominant negative mutant AtAAF^palm^. Proteins were prepared from the 35S::*AtAAF*+*GFP* and 35S::*AtAAF^palm^* +*GFP* plants by the biotin switch palmitoylation assay. Western blot analysis of proteins with the presence (+) and absence (−) of hydroxylamine treatment using a GFP antibody

*AtAAF* transcript was high in flower buds (Figure 2a) and was higher in sepals/petals/stamens during early flower development (stage 11) and in carpels during late flower development (stage 14) (Figure 2b). In AtAAF::*GUS Arabidopsis* flowers, GUS activity was detected in the developing sepals, petals and anthers before stage 10 (Figure 2c,d). GUS activity decreased in the sepals/petals and was absent in the anthers after stage 12 (Figure 2c,e,f). GUS activity was detected in developing ovules of the carpel from stage 12 until the maturation of the siliques (Figure 2e, f).

An *Agrobacterium* infiltration‐mediated transient expression assay was applied in identification of the localization of AtAAF protein. The cDNA of *AtAAF* fused with *GFP* driven by the cauliflower mosaic virus (CaMV) 35S promoter (35S::*AtAAF*+*GFP*) was transformed into *Agrobacterium* and infiltrated into the leaf epidermis of *Nicotiana benthamiana*. The result showed that AtAAF+GFP fusion proteins accumulated at the plasma membrane (Figure 2h). In contrast, GFP proteins accumulated mainly in the cytosol (Figure 2g).

Palmitoylation of tetraspanins in mammalian cells has been reported previously (Charrin et al., 2002; Hua, Green, Wong, Warsh, & Li, 2001; Israels & McMillan‐Ward, 2010; Yang et al., 2002). Similar to *OnAAF* and *PaAAF* in orchids, *AtAAF* is predicted to be a member of the tetraspanin family and contains four palmitoylation sites, C65, C66, C252 and C253 (Figure S1), predicted by CSS‐alm (Ren et al., 2008). It is possible that *AtAAF* may also require palmitoylation to perform its function and that a mutation in the palmitoylation sites may generate a palmitoylation‐deficient mutant for *AtAAF*. Therefore, we generated transgenic plants that ectopically expressed *AtAAF* (35S::*AtAAF*) or a palmitoylation‐deficient mutant form of *AtAAF* (*AtAAF^palm^*) with multiple cysteine point mutations at C65S, C66S, C252S, and C253S (35S::*AtAAF^palm^*) fused with GFP. To determine whether the AtAAF protein was palmitoylated, AtAAF+GFP or AtAAF^palm^+GFP was isolated from 35S::*AtAAF+GFP* or 35S::*AtAAF^palm^+GFP* transgenic plants and a biotin switch assay (Hemsley et al., 2008) was performed using anti‐GFP antibody (Figure 2i). The results indicated that the AtAAF protein was indeed palmitoylated, whereas the AtAAF^palm^ protein was not (Figure 2i).

### 3.4 Ectopic expression of *AtAAF* increases the size of flower organs and seeds

**Figure 3:**
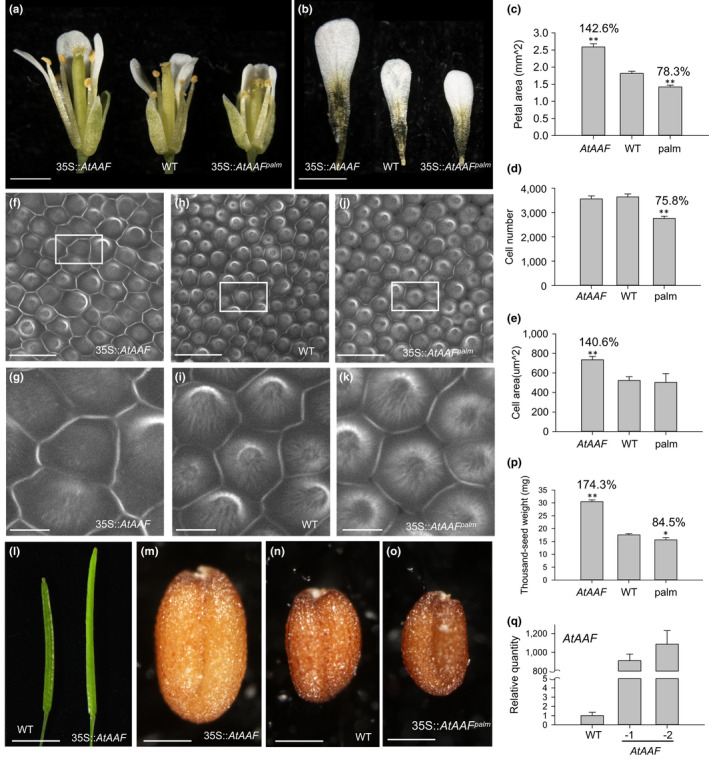
Functional analysis of flower and seed size for 35S::*AtAAF* and 35S::*AtAAF^palm^ Arabidopsis*. (a, b) The flower (a) and petals (b) of 35S::*AtAAF* (left), wild‐type (middle), and 35S::*AtAAF^palm^* (right) Arabidopsis. Bar = 1 mm. (c) Size comparison of the petals from 35S::*AtAAF* (*AtAAF*), WT, and 35S::*AtAAF^palm^* (palm) Arabidopsis. Petals from three flowers for each plant (*AtAAF*, WT, and palm) were used to measure the average size. The size of the wild‐type petals is set at 100%. (d, e) Comparison of total cell number (d) and the epidermal cell size (e) in petals from 35S::*AtAAF* (*AtAAF*), wild‐type (WT), and 35S::*AtAAF^palm^* (palm) Arabidopsis. (f–k) Confocal laser scanning microscopy of the epidermal cells in the petals from 35S::*AtAAF* (f, g), WT (h, i), and 35S::*AtAAF^palm^* (j, k) flowers. (g), (i), and (k) are close‐ups of (f), (h), and (j), respectively. Bar = 20 μm in (f, h, j) and 5 μm in (g, i, k). (l) The mature siliques of 35S::*AtAAF* (right) and wild‐type (WT, left) Arabidopsis. Bar = 5 mm. (m–o) The seeds of 35S::*AtAAF* (m), WT (n), and 35S::*AtAAF^palm^* (o) Arabidopsis. Bar = 0.2 mm. (p) Comparison of the weight for 1,000 seeds from 35S::*AtAAF* (*AtAAF*), WT, and 35S::*AtAAF^palm^* (palm) Arabidopsis. (q) Detection of *AtAAF* expression for one WT and two 35S::*AtAAF* (−1, −2) plant. The asterisks in (c, d, e, p) indicate a significant difference from the WT value (*p ≤ .05, **p ≤ .01). Statistic analysis was measured by Student's *t* test

To investigate function of the *AtAAF* gene, 35S::*AtAAF* transgenic *Arabidopsis* were generated and analyzed. 35S::*AtAAF* transgenic plants showed abnormal phenotypes including sterility (see below) and produced larger flowers than did the wild‐type plants (Figure 3a), increasing petal size by approximately 42% (Figure 3a–c) in transgenic *Arabidopsis*. 35S::*AtAAF* petals contained similar numbers of cells (Figure 3d), but they were 40% larger (Figure 3e–g) than cells in wild‐type petals (Figure 3h,i). These results suggest that *AtAAF* promoted petal cell expansion. The length of mature siliques on 35S::*AtAAF* plants after manual self‐pollination was also increased compared with that on wild‐type siliques (Figure 3l). 35S::*AtAAF* seeds (Figure 3m) were clearly larger and heavier than wild‐type seeds (Figure 3n), increasing by approximately 74% (Figure 3p). Furthermore, the altered phenotype for the 35S::*AtAAF* plants was correlated with high level of *AtAAF *expression (Figure 3q).

### 3.5 Ectopic expression of palmitoylation‐deficient C65S, C66S, C252S, and C253S multiple point mutations in AtAAF causes early senescence and reduced size of flower organs and seeds

To further determine the role of *AtAAF*, *AtAAF* loss‐of‐function T‐DNA insertion lines, SALK_018161 and SALK_115646 (both containing an insertion in the 5′ promoter region) (Figure S5A), were analyzed. The result indicated that these *AAF* mutants were phenotypically indistinguishable from wild‐type plants in both vegetative and reproductive development. Further analysis indicated that the expression of *AAF* in these T‐DNA insertion mutants was completely abolished (Figure S5B). This finding indicates a possible functional redundancy between *AAF* and other unknown genes in Arabidopsis.

Based on the data from mammalian cells, tetraspanins form a tetraspanin‐enriched microdomain (TEM), a tetraspanin web, by interacting with one another and other transmembrane proteins such as integrins and other adhesion receptors (Charrin et al., 2014; Hemler, 2005; Reimann et al., 2017; Zuidscherwoude et al., 2015). The organization of the integrin–tetraspanin microdomain and modulation of adhesion‐dependent signaling were mediated by palmitoylation of tetraspanins (Berditchevski et al., 2002). It has been shown that ectopic expression of the human palmitoylation‐deficient tetraspanin CD151 in Rat‐1 cells impaired the interactions of the endogenous tetraspanins CD63 and CD81 and weakened the association of integrin with the tetraspanin‐enriched microdomains and affected integrin‐dependent signaling (Berditchevski et al., 2002). It also showed that the mutation in palmitoylation site reduced the ability of human tetraspanin CD81 to interact with other proteins (Delandre et al., 2009) and tetraspanin CD82 to inhibit cancer cell migration and invasion (Zhou et al., 2004). These results indicated that overexpression of a palmitoylation‐deficient tetraspanin could occupy the positions in the tetraspanin‐enriched microdomain and impaired the interactions for all other tetraspanins with the redundant function. This resulted in the production of a palmitoylation‐deficient mutant in which the function of all the redundant tetraspanins can be altered in the same time. Thus, a palmitoylation‐deficient *AtAAF* (*AtAAF^palm^*) with multiple cysteine point mutations at C65S, C66S, C252S, and C253S was ectopically expressed in Arabidopsis to generate palmitoylation‐deficient mutant of *AtAAF.*

**Figure 4:**
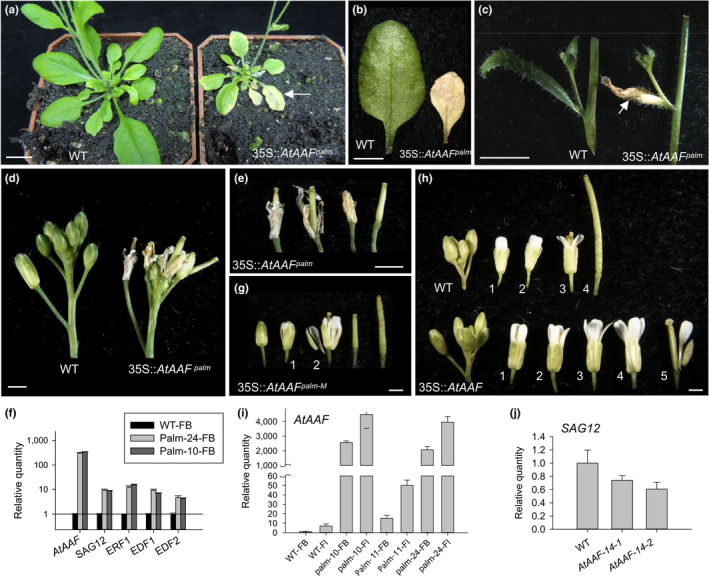
Phenotypic analysis of 35S::*AtAAF^palm^* dominant negative mutant *Arabidopsis*. (a) Five‐week‐old 35S::*AtAAF^palm^* Arabidopsis (right) produced smaller rosette leaves which showed earlier senescence (arrowed) than the wild‐type plants (left). Bar = 10 mm. (b, c) 35S::*AtAAF^palm^* transgenic plants showed earlier senescence of rosette (b, right) and cauline leaves (c, right, arrow) than the wild‐type (WT, left). Bar = 5 mm. (d) Flowers from a severe 35S::*AtAAF^palm^* inflorescence which showed earlier senescence than WT flowers. Bar = 1 mm. (e) Close‐up of the flowers from a severe 35S::*AtAAF^palm^* inflorescence shown in (d). Bar = 1 mm. (f) Detection of expression for *AtAAF,* senescence‐related gene (*SAG12*), and the ethylene response genes (*ERF1, EDF1,* and *EDF2*) in floral buds (FB) before stage 12 from one WT plant and two severe 35S::*AtAAF^palm^* plants (palm‐24 and palm‐10). Transcript levels in 35S::*AtAAF^palm^* plants are presented relative to those in the wild‐type plant, which were set to 1. (g) Close‐up of the flowers from a medium–severe 35S::*AtAAF^palm‐M^* inflorescence. The numbers indicate the positions of the open flowers. Bar = 1 mm. (h) Close‐up of the flowers from wild‐type (top row) and 35S::*AtAAF* (bottom row) inflorescences. The 35S::*AtAAF* flowers are clearly larger and abscised later than wild‐type flowers. The numbers indicate the positions of the open flowers. Bar = 1 mm. (i) Detection of *AtAAF* expression in FB and mature flowers (Fl) of one WT, two severe 35S::*AtAAF^palm^* (palm‐10, ‐24), and one medium–severe 35S::*AtAAF^palm^* (palm‐11) plants. (j) Detection of *SAG12* expression in WT and two 35S::*AtAAF* (*AtAAF‐14‐1* and *AtAAF‐14‐2*) plants

Pronounced small‐leaf and early‐senescence phenotypes were observed in the severe 35S::*AtAAF^palm^* transgenic plants (Figure 4a). Early senescence was observed in the rosette leaves (Figure 4a,b), developing cauline leaves (Figure 4c), and young flower buds (Figure 4d,e) of the severe 35S::*AtAAF^palm^* plants. The senescence‐marker gene *SAG12* (Swartzberg, Dai, Gan, Amasino, & Granot, 2006) and ethylene response genes *EDF1*, *EDF2,* and *ERF1* (Alonso et al., 2003; Stepanova & Alonso, 2005) were clearly up‐regulated in these severe 35S::*AtAAF^palm^* plants (Figure 4f). In the medium–severe 35S::*AtAAF^palm^* transgenic plants, flower senescence was slightly promoted (at number 2–3) (Figure 4g) compared with wild‐type plants (at number 3–4) (Figure 4h). The severity of the phenotype for the 35S::*AtAAF^palm^* plants was correlated with *AtAAF^palm^* expression level (Figure 4i). The early senescence of flowers and the up‐regulation of *SAG12* were opposite to those observed in 35S::*AtAAF* plants (Figure 4h,j).

Interestingly, both flowers (Figure 3a) and petals (Figure 3b) of the medium–severe 35S::*AtAAF^palm^* plants were ~22% smaller than those of wild‐type plants (Figure 3c). The number of epidermal cells in 35S::*AtAAF^palm^* petals was ~24% less than in the wild type (Figure 3d), but the average cell size (Figure 3e) and morphology (Figure 3j,k) were comparable to that in the wild type (Figure 3e,h,i). In addition to petals, seeds produced by medium–severe 35S::*AtAAF^palm^* plants (Figure 3o) were slightly smaller and approximately 15.5% lighter than wild‐type seeds (Figure 3n,p).

### 3.6 Ectopic expression of *AtAAF* shows male sterility and indehiscence of the anther which can be rescued by exogenic jasmonic acid

**Figure 5:**
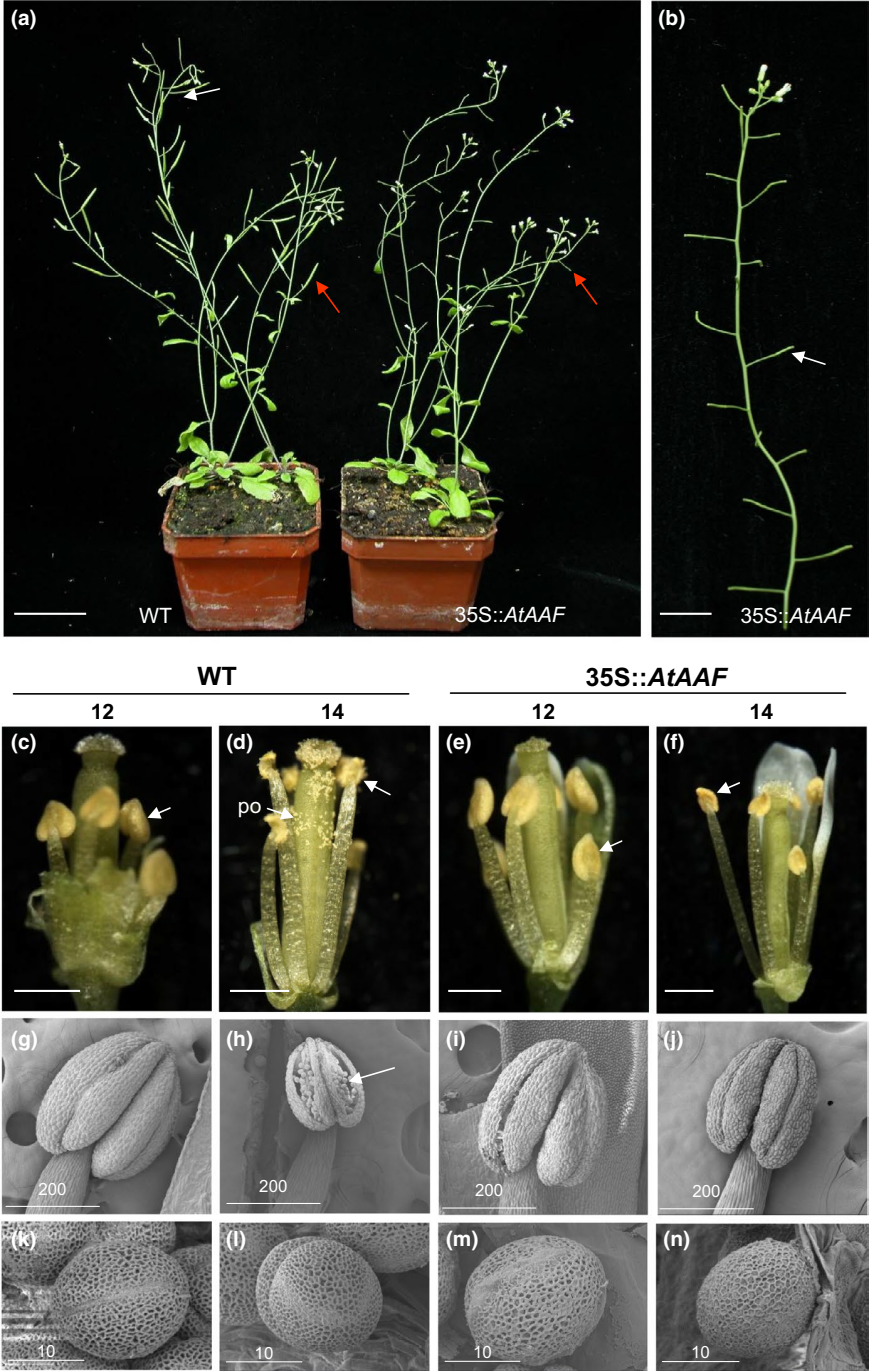
Phenotypic analysis of Arabidopsis plants ectopically expressing *AtAAF*. (a) A 35S::*AtAAF* plant (right) was sterile and produced short siliques (arrow), whereas wild‐type plants (WT, left) produced long, well‐developed siliques (arrow). Bar = 30 mm. (b) Inflorescences from a 35S::*AtAAF* plant that showed sterility with short siliques (arrow). Bar = 10 mm. (c–f) Indehiscent anthers (arrow) were observed at stage 12 in WT plants (c), stages 12 (e), and 14 (f) 35S::*AtAAF* flowers compared with stage 14 wild‐type flower (d), which showed normal anther dehiscence (arrow) and pollen (po) release. Bar = 0.5 mm. (g–j) Close‐up of stage 12 (g) wild‐type, stages 12 (i), and 14 (j) 35S::*AtAAF* anther compared with stage 14 wild‐type anther (h) by SEM, which showed normal anther dehiscence and pollen (arrow) release. Bar = 200 μm. (k–n) Close‐up of the stage 12 (k), 14 (l) wild‐type and stage 12 (m), 14 (n) 35S::*AtAAF* pollen grains by SEM. Bar = 10 µm

35S::*AtAAF Arabidopsis* plants also produced sterile flowers in which the siliques failed to elongate (Figure 5a,b). 35S::*AtAAF* anthers were indehiscent at all stages of flower development (Figure 5e,f). In contrast, wild‐type anthers were completely dehiscent, and pollen was released after stage 14 of flowering (Figure 5c,d). When further examined by SEM, the wild‐type anther dehisced at stage 14 (Figure 5g,h), and the pollen grains that were released from wild‐type anthers exhibited an egg shape, 30 × 5 μm in size (Figure 5k,l). When the indehiscent anthers from the stage 12 (Figure 5i) and 14 (Figure 5j) flowers of the 35S::*AtAAF* plants were opened manually and compared with wild‐type pollen, normal pollen grains were observed (Figure 5m,n).

Further examination of the pollen by Alexander's stain, which can distinguish viable pollen from nonviable pollen (Alexander, 1969), normal viability (dark red staining) similar to that of wild‐type pollen (Figure S6A) was observed in the 35S::*AtAAF* pollen (Figure S6B). The pollen from 35S::*AtAAF* flowers was viable, as silique elongation and seed maturation were observed (Figure S6C,D) after the 35S::*AtAAF* flowers were manually self‐pollinated. The 35S::*AtAAF* pistil was normal, as fully developed siliques with normal seed development (Figure S6E,F) were observed after manual pollination with wild‐type pollen.

One of the reasons for defects in anther dehiscence is the mutation of JA biosynthesis genes such as *DAD1* and *OPR3* (Ishiguro, Kawai‐Oda, Ueda, Nishida, & Okada, 2001; Sanders et al., 2000). We thus investigated whether an external application of JA rescued the sterility of the 35S::*AtAAF* plants in a similar manner to that in JA‐treated *dad1* flowers (Ishiguro et al., 2001). Dehiscence of anthers (Figure S6G) and elongation of siliques (Figure S6I) were observed in JA‐treated 35S::*AtAAF* flowers. These phenotypes were clearly distinguished from those observed in the JA‐untreated 35S::*AtAAF* flowers, which did not show anther dehiscence (Figure S6H) or silique elongation (Figure S6I). Further analysis indicated that the expressions of genes involved in JA biosynthesis, such as *DAD1* and *OPR3*, were all significantly down‐regulated in the flowers of 35S::*AtAAF* plants (Figure S6J).

### 3.7 The secondary wall thickness is deficient and the expression of *NST1/2* that participate in lignin accumulation of anther secondary wall is down‐regulated in 35S::*AtAAF* transgenic *Arabidopsis*

In wild‐type plants, secondary thickening occurs in the endothecium before anther dehiscence, and the surrounding cell layers of the anther do not undergo secondary thickening (Cecchetti et al., 2013; Yang et al., 2007). To examine the formation of secondary wall thickness, cellulose staining with calcofluor white and lignin staining with auramine O were performed in the endothecium of the developing anther in 35S::*AtAAF* and wild‐type plants. Secondary thickening in the endothecium was observed in wild‐type anthers at stage 11 (Figure S7A–C), just prior to anther dehiscence, as well as at stage 13 (Figure S7D–F), while the anthers dehisced completely. In contrast, no secondary thickening or lignification was observed in the anther endothecium of 35S::*AtAAF* plants at either stage 11 (Figure S7G–I) or stage 13 (Figure S7J–L). When the expression of *MYB26*, *NST1,* and *NST2*, which are necessary for secondary wall thickening in the anther wall (Mitsuda, Seki, Shinozaki, & Ohme‐Takagi, 2005; Steiner‐Lange et al., 2003), was examined, a clear down‐regulation for these genes was observed in 35S::*AtAAF* transgenic plants (Figure S7M). This result clearly indicates that altered anther dehiscence in 35S::*AtAAF* plants is correlated with altered expression of genes participating in the regulation of secondary wall thickening in anthers.

### 3.8 Ectopic expression of *AtAAF* enhances drought/salt tolerance and auxin response

**Figure 6:**
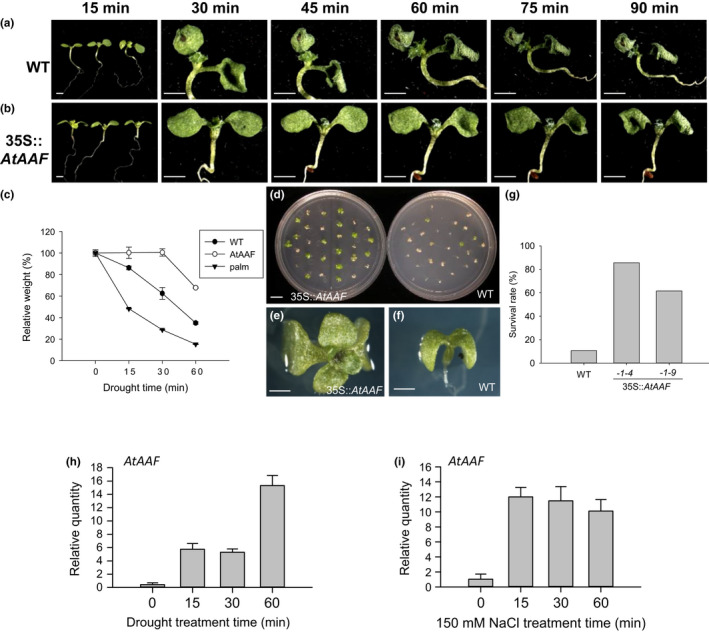
The analysis of drought and salt tolerance for 35S::*AtAAF* plants. (a, b) One‐week‐old wild‐type (a) and 35S::*AtAAF* (b) seedlings were tested for drought tolerance by removing them from MS medium and exposing them to a stream of air for 15, 30, 45, 60, 75, or 90 min. Bar = 5 mm. (c) The 35S::*AtAAF* (*AtAAF*) seedlings clearly withered later and lost less weight than wild‐type (WT) seedlings at 15, 30, and 60 min after drought treatment. In contrast, the 35S::*AtAAF^palm^* (palm) seedlings clearly withered earlier and lost more weight than WT seedlings at 15, 30, and 60 min after drought treatment. (d) Comparison of germination for the 35S::*AtAAF* and WT seedlings grown on MS medium containing 150 mM NaCl. Bar = 10 mm. (e, f) Close‐up of the 35S::*AtAAF* (e) and WT (f) seedlings grown on MS medium containing 150 mM NaCl. Bar = 5 mm. (g) The survival rate for WT and two lines of 35S::*AtAAF (‐1‐4, ‐1‐9*) seedlings grown on MS medium containing 150 mM NaCl. (h, i) Detection of *AtAAF* expression after 15, 30, or 60 min of drought (h) or salt (i) treatment in wild‐type plants

35S::*AtAAF* expression also enhanced the drought and salt tolerance of transgenic *Arabidopsis*. One‐week‐old wild‐type seedlings clearly withered 15–30 min after they were removed from the MS medium and exposed to a stream of air (Figure 6a) and subsequently lost approximately 20%–40% of their weight (Figure 6c). By contrast, the 35S::*AtAAF* seedlings did not show signs of wilt 30 min after treatment, and their weight was similar to that of untreated seedlings (Figure 6b,c). The 35S::*AtAAF* seedlings started to show signs of wilt 60 min after treatment, and they lost approximately 30% of their weight (Figure 6b,c). During this same time, wild‐type seedlings severely withered and lost approximately 70% of their weight (Figure 6a,c). Unlike the 35S::*AtAAF* plants, the 35S::*AtAAF^palm^* palmitoylation‐deficient mutant seedlings clearly showed lower drought tolerance, withered earlier and lost more weight than wild‐type seedlings from 15 to 60 min following drought treatment (Figure 6c). The wild‐type seeds barely germinated into seedlings (Figure 6d,f) and had a survival rate as low as 10% (Figure 6g) in MS medium containing 150 mM NaCl. By contrast, more than 60%–80% of the 35S::*AtAAF* seeds germinated and produced leaves (Figure 6d,e,g). Unsurprisingly, a clear up‐regulation of *AtAAF* expression was observed 15 min after drought or salt treatments (Figure 6h,i).

**Figure 7:**
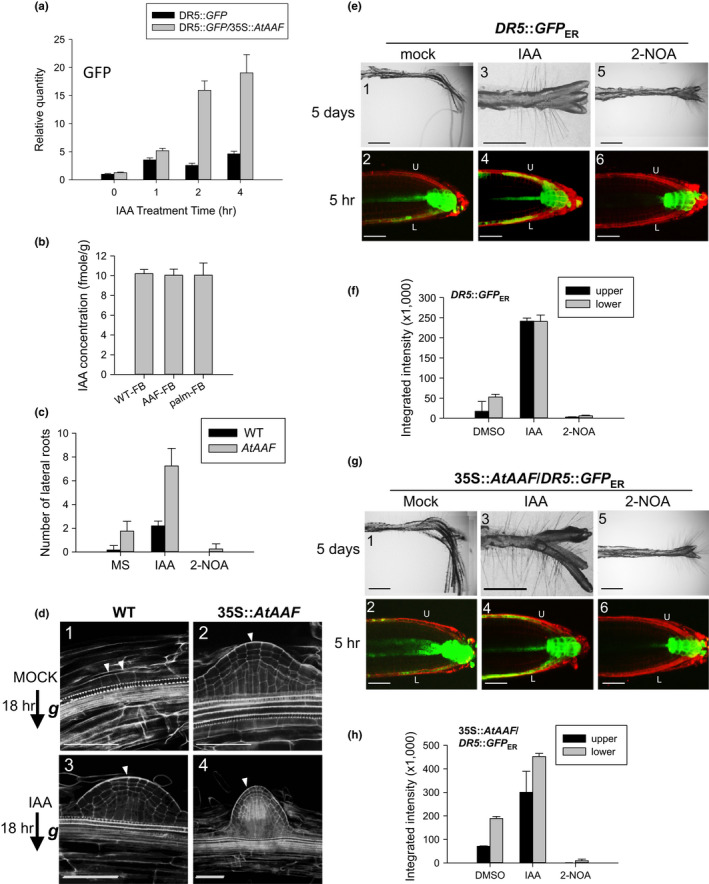
Investigation of the relationship between *AtAAF* and Auxin response in *Arabidopsis*. (a) Detection of *GFP* expression in DR5::*GFP* and DR5::*GFP*/35S::*AtAAF* transgenic Arabidopsis after 1, 2, and 4 hr of external IAA treatment. (b) Detection of the amount of IAA in wild‐type (WT), 35S::*AtAAF* (*AAF*), and 35S::*AtAAF^palm^* (palm) flower buds (FB). (c) Number of lateral roots produced in WT and 35S::*AtAAF* (*AtAAF*) Arabidopsis seedlings grown on MS medium with or without IAA/2‐NOA (auxin influx inhibitor) treatments. (d) The development of the primordia (arrowhead) for lateral roots of the WT and 35S::*AtAAF* roots of 14 DAG seedlings 18 hr post‐gravitropic induction with (d‐3 and d‐4) or without (d‐1 and d‐2) IAA treatment. Stage I (d‐1), VII (d‐2), VI (d‐3), and beyond stage VIII (d‐4) primordia of lateral roots were formed. Bar = 50 μm. (e) Root gravitropic assays for DR5::*GFP* roots with 5 days of a 90‐degree gravitropic stimulus without (mock, e‐1) or with IAA (e‐3) or 2‐NOA (e‐5) treatment. Bar = 0.5 mm. The detection of GFP in the lower (L) and upper (U) parts of the DR5::*GFP* root tips 5 hr after 90‐degree gravitropic stimulus without (mock, e‐2) or with IAA (e‐4) or 2‐NOA (e‐6) treatment. Bar = 50 μm. (f) The detection of GFP integrated intensity in lower and upper parts of the DR5::*GFP* root tips from (e‐2), (e‐4) and (e‐6). (g) Root gravitropic assays for 35S::*AtAAF*/DR5::*GFP* roots after 5 days of a 90‐degree gravitropic stimulus without mock (g‐1) or with IAA (g‐3) or 2‐NOA (g‐5) treatment. Bar = 0.5 mm. The detection of GFP in lower (L) and upper (U) parts of the 35S::*AtAAF*/DR5::*GFP* root tips 5 hr after 90‐degree gravitropic stimulus without (mock, g‐2) or with IAA (g‐4) or 2‐NOA (g‐6) treatment. Bar = 50 μm. (h) The detection of GFP integrated intensity in lower and upper parts of the 35S::*AtAAF*/DR5::*GFP* root tips from (g‐2), (g‐4), and (g‐6)

It has been reported that auxin regulates flower organ development, since the elongation of petals and stamens is defective in *auxin response factor 6* (*arf6*) and *arf8* double‐mutant plants (Tabata et al., 2010). Auxin also regulates anther dehiscence by controlling the timing of endothecium secondary cell wall lignification and JA biosynthesis (Cecchetti et al., 2013), and auxin is involved in the response to abiotic stresses such as drought and salt (Li et al., 2015; Shi et al., 2014). Since 35S::*AtAAF* altered flower organ size, anther dehiscence, and the response to drought and salt stresses, *AtAAF* may function in auxin regulation. To investigate this assumption, 35S::*AtAAF* was introduced into DR5::*GFP Arabidopsis*, which contains a highly active synthetic auxin response element DR5 with a GFP reporter. The level of GFP transcript was clearly up‐regulated after 1–4 hr of external IAA treatment in both DR5::*GFP* and DR5::*GFP*/35S::*AtAAF* plants (Figure 7a). Interestingly, the increased amount of GFP transcript was much higher in DR5::*GFP*/35S::*AtAAF* than in DR5::*GFP* plants after IAA treatment (Figure 7a). A similar amount of IAA was detected in wild‐type, 35S::*AtAAF,* and 35S::*AtAAF^palm^* plants (Figure 7b), suggesting that 35S::*AtAAF* enhanced efficiency of the auxin response rather than increasing auxin production.

Since auxin is a key signal that coordinates lateral root primordia outgrowth (Benková et al., 2003; Péret et al., 2012; Swarup & Bennett, 2009), lateral root production was measured in 2‐week‐old wild‐type and 35S::*AtAAF* seedlings. The results indicated that 35S::*AtAAF* plants produced significantly more lateral roots than did wild‐type plants (Figure 7c). Furthermore, IAA‐treated 35S::*AtAAF* plants produced three times more lateral roots than did IAA‐treated wild‐type plants (Figure 7c). This result supports an enhanced auxin response in 35S::*AtAAF* that could stimulate the formation of lateral roots. Formation of lateral roots was significantly prohibited in 35S::*AtAAF* and wild‐type seedlings grown at medium containing 2‐NOA (auxin influx inhibitor) as controls (Figure 7c).

### 3.9 Lateral root primordia of 35S::*AtAAF* develops faster than wild type

It has been reported that a 90‐degree gravitropic stimulus can induce lateral root primordia (LRP) to form on the outer side of bending roots (Lucas, Godin, Jay‐Allemand, & Laplaze, 2008; Péret et al., 2009). In addition, auxin plays a key role as a signal that coordinates lateral root primordia outgrowth, outer tissue deformation, and cell separation (Benková et al., 2003; Lucas et al., 2008; Swarup & Bennett, 2009). There are eight LRP stages previously described (Péret et al., 2012). When grown on a 90‐degree gravitropic stimulus, wild‐type seedlings produced stage I (Figure 7d‐1) lateral root primordia 18 hr post‐gravitropic induction (pgi), while 35S::*AtAAF* seedlings produced stage VII LRP (Figure 7d‐2) in the same time frame. Following auxin treatment, the wild‐type seedlings developed stage VI LRP (Figure 7d‐3), while the LRP produced by 35S::*AtAAF* seedlings were beyond stage VIII (Figure 7d‐4) at 18 hr pgi. These results again suggest that 35S::*AtAAF* could enhance the auxin response and hasten the formation of LRP on the roots.

### 3.10 35S::*AtAAF* root tip retains gravitropism under auxin treatment

The movement of auxin in gravitropic response in roots is well understood (Chen, Rosen, & Masson, 1999). Exogenous auxin treatment on roots disrupts the endogenous auxin gradient and prevents gravitropism (Chen et al., 1999; Ishikawa & Evans, 1995). Auxin‐induced asymmetric gravitropism was observed in DR5::*GFP* transgenic roots after 5 days of a 90‐degree gravitropic stimulus (Figure 7e‐1). GFP intensity was greater in the lower parts than in the upper parts of DR5::*GFP* root tips 5 hr after 90‐degree gravitropic stimulus (Figure 7e‐2,f). The reaction to this asymmetric gravitropism was impaired in DR5::*GFP* transgenic roots 5 days after auxin treatment (Figure 7e‐3) due to the equal distribution of GFP in the lower and upper parts of the auxin‐treated DR5::*GFP* root tips 5 hr after 90‐degree gravitropic stimulus (Figure 7e‐4,f). As expected, the GFP signal was clearly higher in auxin‐treated than in untreated DR5::*GFP* root tips (Figure 7f).

Similar to DR5::*GFP* transgenic roots, the auxin‐induced asymmetric gravitropism was also retained in DR5::*GFP*/35S::*AtAAF* double transgenic roots under the same treatment (Figure 7g‐1). GFP fluorescence was also brighter in the lower parts of the DR5::GFP/35S::*AtAAF* root tips than in the upper parts (Figure 7g‐2,h). Unlike DR5::*GFP* transgenic roots, asymmetric gravitropism was still retained in DR5::*GFP*/35S::*AtAAF* double transgenic roots 5 days after auxin treatment (Figure 7g‐3). GFP intensity was higher in the lower parts than in the upper parts of the auxin‐treated DR5::*GFP*/35S::*AtAAF* root tips 5 hr after 90‐degree gravitropic stimulus (Figure 7g‐4,h) and was also higher in auxin‐treated than in untreated *DR5*::GFP/35S::*AtAAF* root tips (Figure 7h). Furthermore, the GFP signal was higher in DR5::*GFP*/35S::*AtAAF* than in DR5::*GFP* either with or without IAA treatment (Figure 7f,h). In controls, asymmetric gravitropism was absent in both DR5::*GFP* and DR5::*GFP*/35S::*AtAAF* transgenic roots 5 days after 2‐NOA (auxin influx inhibitor) treatment (Figure 7e‐5,g‐5). GFP was undetectable in both the lower and upper parts of the 2‐NOA‐treated DR5::*GFP* (Figure 7e‐6) and DR5::*GFP*/35S::*AtAAF* (Figure 7g‐6) root tips 5 hr after 90‐degree gravitropic stimulus.

## 4 DISCUSSION

In this study, orthologs of the tetraspanin gene *AAF,* which are associated with the regulation of perianth size, were identified and characterized in *Oncidium* and *Phalaenopsis* orchids. The results obtained indicate that the higher the expression level of orchid *AAF* orthologs, the larger size of the perianth organ produced. In *Oncidium* orchids, after perianth identity was determined by P‐code complexes, *OnAAF* was expressed at a higher level in lips than in sepals/petals and was associated with the production of larger lips. By contrast, *PaAAF* was more highly expressed in sepals/petals than in lips and was associated with the production of relatively small lips in *Phalaenopsis* orchids. This assumption was further supported by functional analysis of orchid *OAGL6‐2* orthologs, a key component in L complex of P code (Hsu et al., 2015), through VIGS experiments. VIGS of *OAGL6‐2* resulted in the production of smaller lips and down‐regulation of *Oncidium OnAAF*. By contrast, VIGS of *PeMADS9* (an *OAGL6‐2* ortholog) resulted in enlarged lips and up‐regulation of *Phalaenopsis PaAAF*. Our findings clearly reveal that although *Oncidium* and *Phalaenopsis* orchids do take different approaches to determining lip and sepal/petal size after P‐code complexes are established, the mechanism and the genes involved are the same. The rule is whenever bigger perianth organs are made in orchids, higher tetraspanin *OnAAF*/*PaAAF* expression is associated. Thus, high *OnAAF* expression in lips is associated with the production of large size of lips in *Oncidium* orchid whereas high *PaAAF* expression in petals is associated with the production of large size of petals in *Phalaenopsis* orchid after their organ identity was determined by P‐code complexes.

One interesting and critical issue that must be explored is the exact role of *AAF* orthologs in positively regulating perianth size and other plant developmental processes. The answer is largely revealed by the analysis of *Arabidopsis AAF* ortholog *AtAAF*. It is interestingly to note that *AtAAF* has been reported to be possibly regulated by MADS box gene *AGL15* in Arabidopsis (Wang et al., 2015). Two putative MADS protein binding site of CArG boxes consensus sequence (CC(A/T)_6_GG) were identified in the promoter region of *AtAAF* (Figure S8). These results indicated that *AAF* orthologues could be regulated by MADS box genes in plants. Thus, it supported the assumption that MADS protein *OAGL6‐2* orthologues may be able to regulate *AAF* orthologues in orchids after P code established.

**Figure 8:**
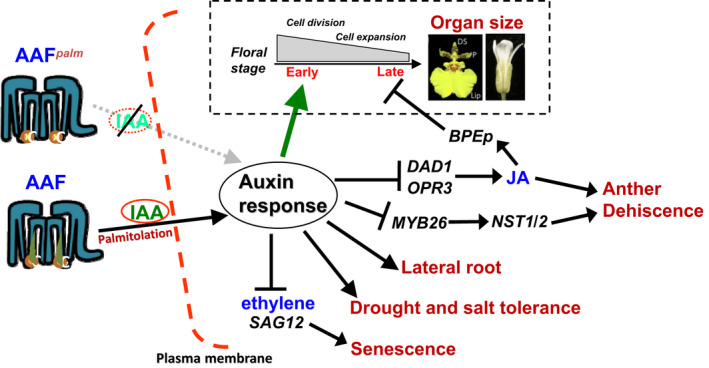
Model for the function of *AAF* orthologues in regulating auxin response and development in plants*.* In plants, the targeting of palmitoylated AAF proteins to the plasma membrane promotes auxin uptake and enhances (→) the auxin response. In flowers, *AAF* is more highly expressed in early than in late developmental stages (gray bar). Thus, it mainly enhances the auxin response and promotes (green arrow) cell division/expansion in the flower organs at an early stage. Ectopic expression of *AAF* extends its affect to the whole flower and results in an increase in the size of the flower organs. By contrast, a palmitoylation‐deficient mutant of AAF reduces ([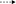]) the auxin response and alters its ability to promote cell division/expansion in early flowering stages resulting in a decrease in flower organ size. In addition, the enhancement of the auxin response by ectopic expression of *AAF* could suppress anther dehiscence during whole flower development by suppressing the expression of *MYB26*/*NST1/2, DAD1*/*OPR3,* and JA activity (which also causes the suppression of the *BPEp* and resulted in the expansion of the flower organs). The enhancement of the auxin response by AAF also suppresses ethylene signaling and organ senescence, promotes drought/salt tolerance and lateral root formation, and retains root tip gravitropism in plants. Dominant negative mutation of *AAF* suppresses auxin response and causes the opposite effect on the processes described above

Not surprisingly, ectopic expression of *AtAAF* increased the size of the flower organs in transgenic *Arabidopsis*. This result revealed a conserved role of the tetraspanin gene *AAF* in the regulation of flower perianth size in monocot orchids and dicot. 35S::*AtAAF* petals contained similar numbers of cells but were 40% larger than cells in wild‐type petals, suggesting that *AtAAF* enlarged petal size primarily by promoting petal cell expansion. In addition, 35S::*AtAAF* also showed a phenotype of male sterility and indehiscence of the anther resembling that found in plants with mutations in genes that participate in JA biosynthesis. The possible involvement of *AtAAF* in regulating JA activity was further evidenced by exogenic JA rescue of the anther indehiscence phenotype and the down‐regulation of genes (*DAD1* and *OPR3*) that participate in JA biosynthesis in 35S::*AtAAF* transgenic *Arabidopsis*. It is interesting to explore the linkage between the regulation of petal size and JA activity controlled by *AtAAF.* Mutation in *OPR3* produced large petals by increasing cell size at developmental stage 14, indicating that JA regulates petal size by suppressing cell expansion at late stages (Brioudes et al., 2009). Thus, *AtAAF* likely controls petal size and anther dehiscence simultaneously by negatively regulating JA activity. This assumption can be supported by the expression pattern of *AtAAF* that is predominantly expressed in sepals/petals/stamens during early flower development. *AtAAF* might suppress JA activity and prohibit early anther dehiscence and cell expansion at these early stages of flower development. When *AtAAF* expression significantly decreased after stage 12, JA activity was promoted, resulting in anther dehiscence and the suppression of petal growth by activating genes, such as *BPEp* (Figure S9), that are involved in limiting petal size by suppressing postmitotic cell expansion (Varaud et al., 2011) (Figure 8). In 35S::*AtAAF Arabidopsis*, the activity of JA was suppressed due to the high level of *AtAAF* expression during all stages of flower development, subsequently causing anther indehiscence throughout flower development and an increase in petal size by promotion of cell expansion due to the suppression of *BPEp* gene (Figure S9) during late flower development. Since *AtAAF* is highly expressed in early developmental stage and may be involved in controlling cell division, ectopic expression of *AtAAF* by 35S::*AtAAF* during early stage should not affect the function of the *AtAAF* in promoting cell division during early developmental stage. Thus, the cell number will not be affected in 35S::*AtAAF* flowers as seen in our result.

In contrast, in the *AtAAF^palm^* palmitoylation‐deficient mutant, *AtAAF* was suppressed throughout flower development, resulting in the suppression of the cell division during early flower development. No or low effect on cell expansion was observed during late flower development since *AtAAF* is normally expressed low during late developmental stage (Figure 8). This caused the production of small petal with fewer cells of normal size in *AtAAF^palm^* palmitoylation‐deficient mutant when compared to those in wild‐type petals.

The next question we propose is in what mechanisms *AtAAF* participates in regulating JA activity. It is worth noting that auxin could inhibit the expression of JA biosynthesis genes *DAD1* and *OPR3* to result in anther indehiscence (Cecchetti et al., 2013). In addition, petal size was increased in *opr3* mutants (Brioudes et al., 2009) and was significantly reduced in *arf6*/*arf8* double‐mutant plants (Tabata et al., 2010). These results suggest a possibly functional correlation between *AtAAF* and auxin in regulating JA activity. Interestingly, auxin‐controlled phenotypes, such as the enhancement of drought and salt tolerance, the development of fast growing lateral root primordia, and the production of significantly more lateral roots, were also observed in 35S::*AtAAF* plants. Thus, we propose that *AtAAF* is likely participating in auxin regulation by either increasing auxin production or enhancing the efficiency of the auxin response. It is clear that a similar amount of IAA was detected in wild‐type, 35S::*AtAAF,* and 35S::*AtAAF^palm^* plants, indicating that *AtAAF* is likely functioning to enhance the efficiency of the auxin response rather than to increase auxin production. This conclusion is supported by three lines of evidence. First, exogenous auxin treatment increased the amount of GFP transcript much higher in DR5::*GFP*/35S::*AtAAF* than in DR5::*GFP* plants. Second, 35S::*AtAAF* plants produced three times more lateral roots than did wild‐type plants, and the formation of LRP on the roots was much faster in 35S::*AtAAF* than in wild‐type seedlings after auxin treatment. Third, after auxin treatment, unlike the asymmetric gravitropism impairment in DR5::*GFP* roots, asymmetric gravitropism was still retained in DR5::*GFP*/35S::*AtAAF* roots. All these results indicate that *AtAAF* could function to increase efficiency of the auxin response, directly or indirectly, in controlling JA activity and plant developmental processes.

As illustrated in Figure 8, our results reveal a possible model for the interaction of the tetraspanin *AAF* orthologues and auxin in regulating plant growth and development. In *Arabidopsis*, the targeting of palmitoylated AtAAF proteins to the plasma membrane promotes auxin uptake and enhances the auxin response, which may affect signaling by other plant hormones (JA, ethylene) and control several developmental processes, such as size of flower organs and seeds, anther dehiscence, lateral root formation, and root tip gravitropism, drought and salt tolerance, and organ senescence. Ectopic expression of *AtAAF* enhanced the auxin response, whereas a palmitoylation‐deficient mutation of *AtAAF* suppressed the auxin response and the processes described above. In *Oncidium* orchids, after perianth identity was determined by P‐code complexes, *OnAAF* was expressed at a higher level in lips than in sepals/petals, enhancing auxin response and subsequently promoting cell division/expansion in lips more than in sepals/petals. This was associated with the production of larger lips. By contrast, *PaAAF* was more highly expressed in sepals/petals than in lips, which was associated with the production of relatively small lips in *Phalaenopsis* orchids. To further unravel the molecular role for *AAF* orthologues in auxin response in the future, the analysis of protein–protein interaction between AAF and the transporters of auxin such as PIN proteins or AUX proteins is necessary. The analysis of signal transduction of cells via AAF from the plasma membrane to nucleus is also needed.

## CONFLICT OF INTEREST

The authors declare no conflict of interest associated with the work described in this manuscript.

## AUTHOR CONTRIBUTIONS

C‐H. Y. developed the overall strategy, designed experiments, and coordinated the project. W‐H. C. performed orchid genes cloning, expression analyses, VIGS experiments, and all the Arabidopsis *AtAAF* experiments. H‐F. H. performed the cryo‐scanning electron microscopy and orchid gene expression analyses. C‐H. Y. collected the orchid samples. W‐H. H. performed the confocal analysis. C‐H. Y. prepared and revised the manuscript.
