# Supplementary material for: A tetraspanin gene regulating auxin response and affecting orchid perianth size and various plant developmental processes
Source: Plant Direct. 2019 Aug 5;3(8):e00157. doi: 10.1002/pld3.157 (PMC6680136; doi:10.1002/pld3.157)
Supplement: Supplementary file 1 [file PLD3-3-e00157-s002.pdf]

Supplemental Figure 1

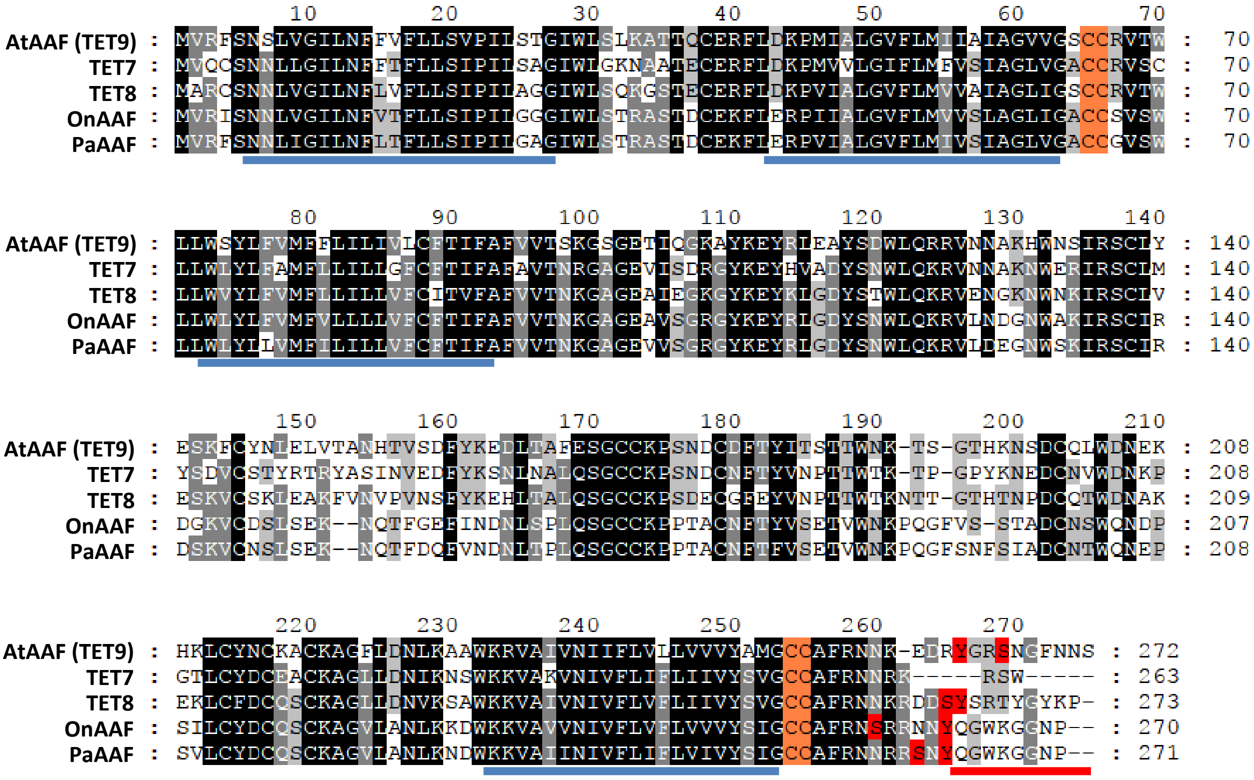

Supplemental Figure 1. Comparison of the protein sequences of AAF orthologues. Comparison of the protein sequences of tetraspanins AtAAF (TET9) (At4g30430), TET7 (At4g28050), TET8 (At2g23810) (*Arabidopsis*), OnAAF (MK674051) (*Oncidium*) and PaAAF (MK674050) (*Phalaenopsis)*. The amino acid residues conserved in AtAAF are highlighted in black; amino acid residues similar to AtAAF are highlighted in gray. The four transmembrane domains are underlined in blue. The palmitoylation sites are indicated by orange boxes and the predicted phosphorylation sites are indicated by red.

Supplemental Figure 2

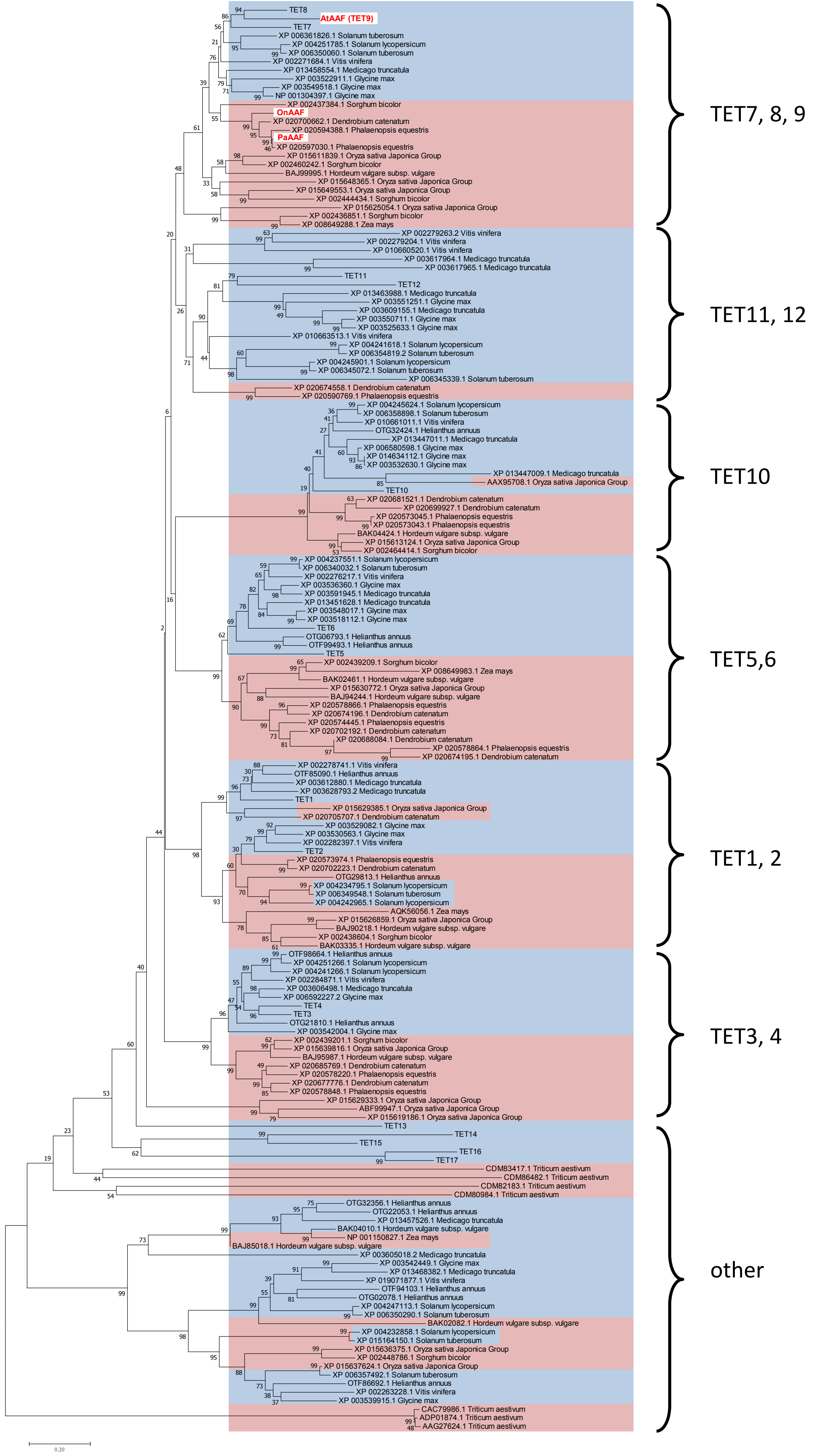

Supplemental Figure 2. Phylogenetic analysis of tetraspanin proteins in plants. Based on the amino acid sequence of the full-length protein, AtAAF (TET9) of Arabidopsis, OnAAF and PaAAF of orchids were assigned to the "TET7/8/9" group of tetraspanin genes. Monocot genes are in pink boxes whereas dicot genes are in blue boxes.

Supplemental Figure 3

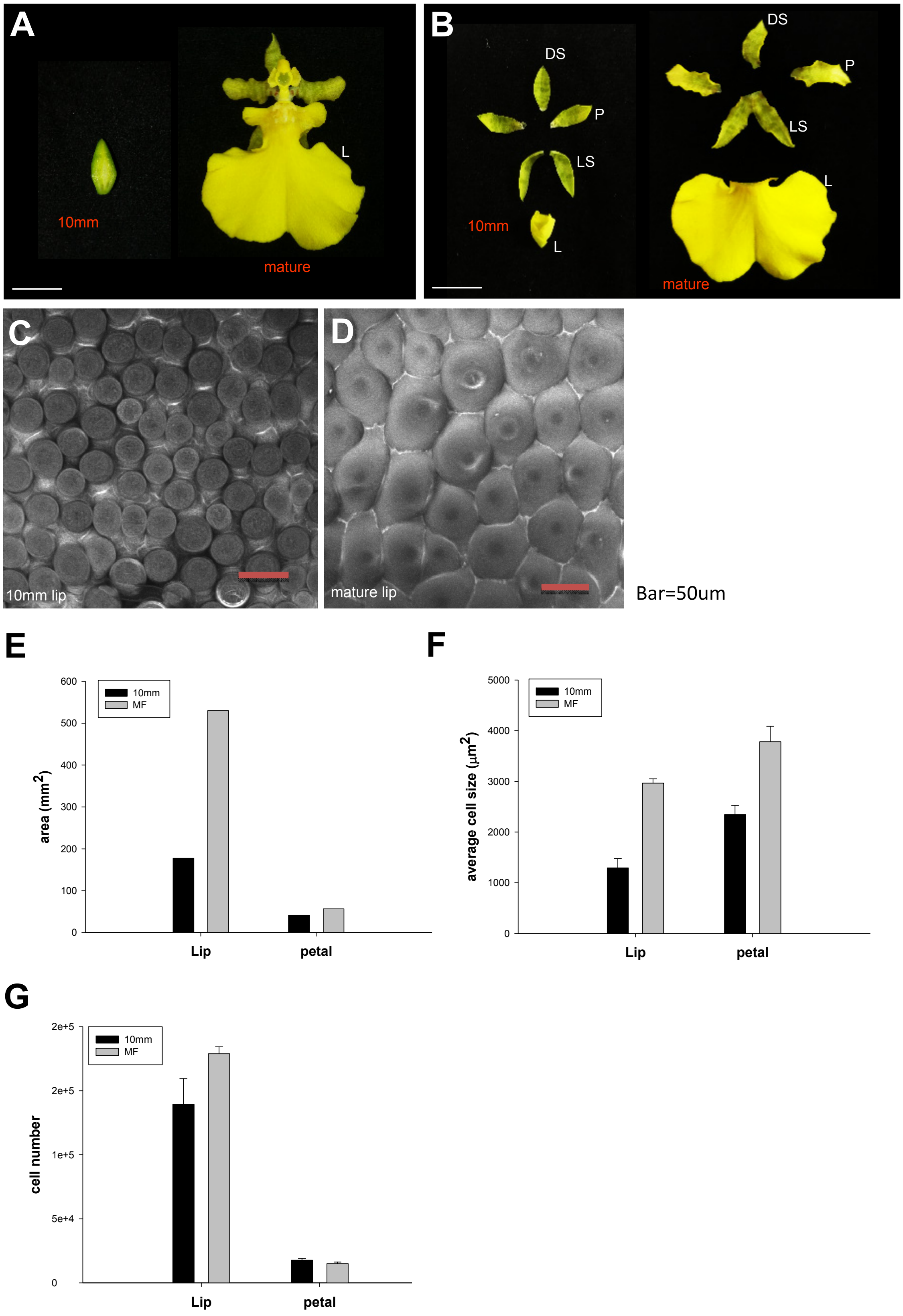

Supplemental Figure 3. The analysis of *Oncidium* flower.
A, Flowers of *O.* Gower Ramsey at different developmental stages (10-mm-long flower bud and mature flower). Bar = 10 mm.
B, Dissection of the perianth organs for a flower bud and a mature flower from (A). *O.* Gower Ramsey flower consists of one dorsal sepal (DS), two lateral sepals (LS), two petals (P) and a lip (L). Lip is smaller in 10-mm-long flower bud than in mature flower. Bar = 10 mm.
C to D, Confocal laser scanning microscopy of the epidermal cells in the lip from 10-mm-long flower bud (C) and mature flower (D). Bar = 50 μm.
E, Size comparison of the lip and petal from 10-mm-long flower bud and mature flower of *Oncidium*.
F to G, Comparison of the epidermal cell size (F) and total cell number (G) in the lip and petal from 10-mm-long flower bud and mature flower of *Oncidium*.

Supplemental Figure 4

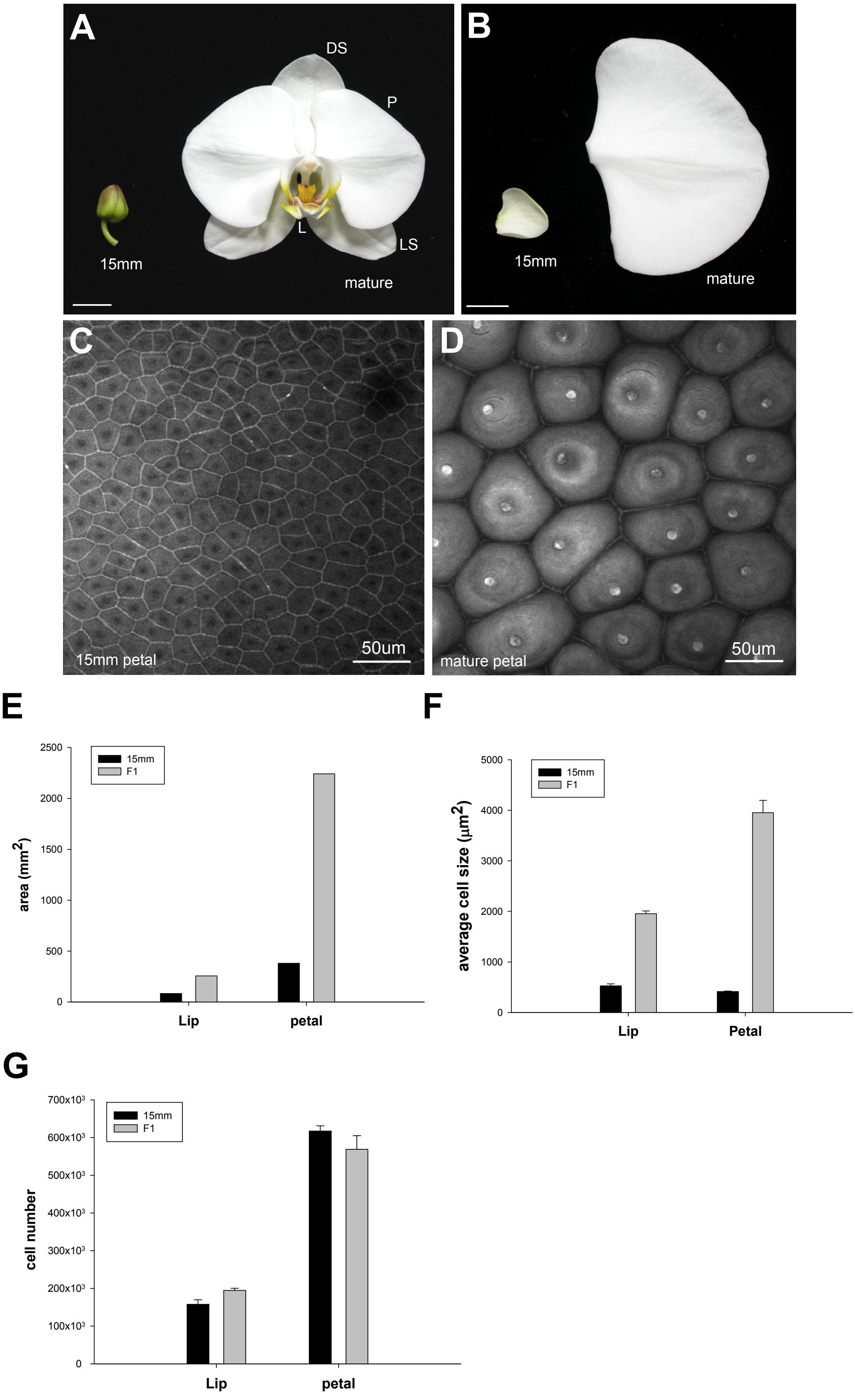

Supplemental Figure 4. The analysis of *Phalaenopsis* flower.
A, Flowers of *Phalaenopsis* Sogo Yukidian 'V3' at different developmental stages (15-mm-long flower bud and mature flower). *Phalaenopsis* flower consists of one dorsal sepal (DS), two lateral sepals (LS), two petals (P) and a lip (L). Bar = 15 mm.
B, Size comparison of the petal from 15-mm-long flower bud and mature flower from (A). Petal is smaller in 15-mm-long flower bud than in mature flower. Bar = 10 mm.
C to D, Confocal laser scanning microscopy of the epidermal cells in the petal from 15-mm-long flower bud (C) and mature flower (D). Bar = 50 μm.
E, Size comparison of the lip and petal from 15-mm-long flower bud and mature flower of *Phalaenopsis*.
F to G, Comparison of the epidermal cell size (F) and total cell number (G) in the lip and petal from 15-mm-long flower bud and mature flower of *Phalaenopsis*.

Supplemental Figure 5

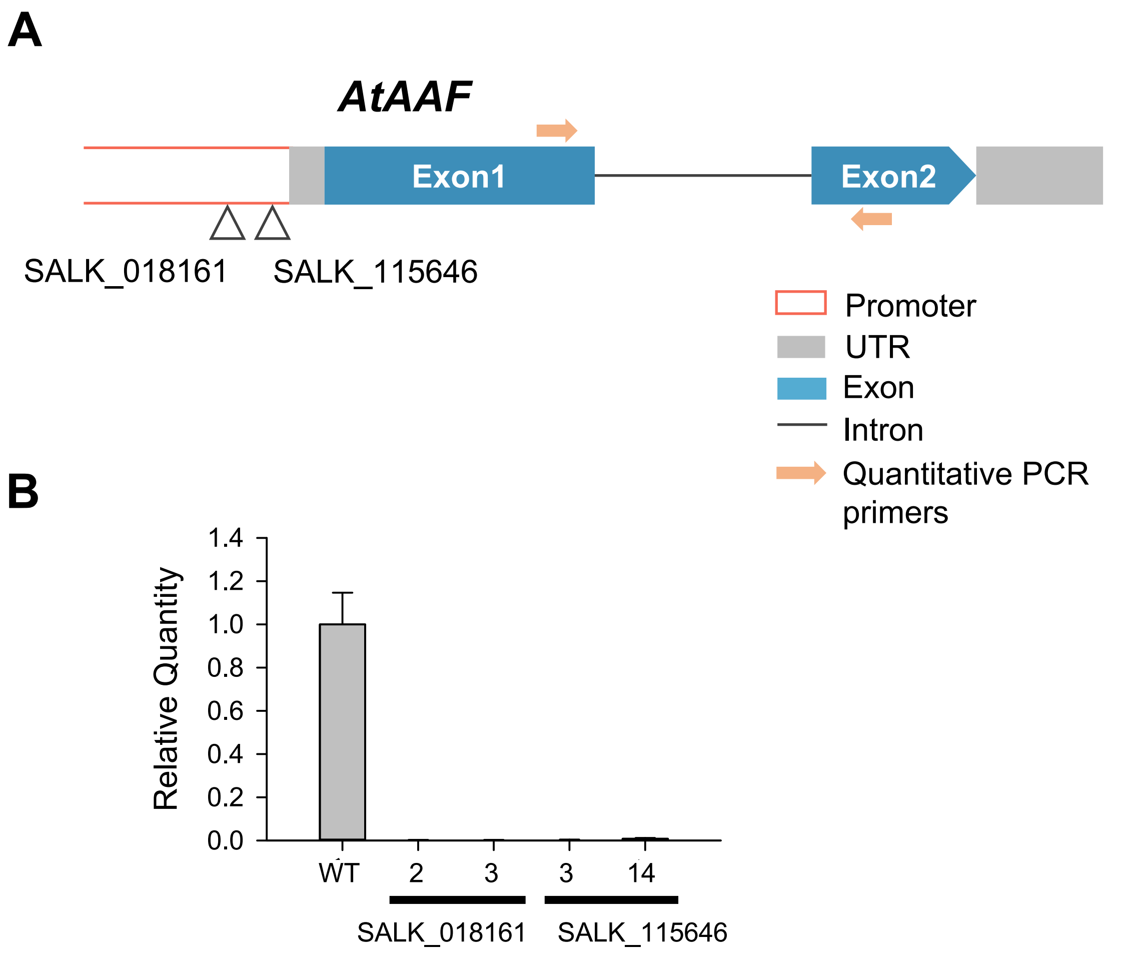

Supplemental Figure 5. Detection of gene expression in *AtAAF* T-DNA insertion lines SALK_018161 and SALK_115646.

A, Genomic region of *AtAAF*. Gray boxes and blue boxes represent UTR and exons for *AtAAF* cDNA, respectively. The location of T-DNA insertion in promoter region of *AtAAF* gene in SALK_018161 and SALK_115646 mutants were indicated as triangles. The position of the primers used to detect the *AtAAF* expression were indicated as orange arrows.

B, Detection of *AtAAF* expression in SALK_018161 (#2, 3) and SALK_115646 (#3, 14) mutants and wild-type (WT) Arabidopsis. mRNA accumulation for *AtAAF* was determined by real-time quantitative PCR. Gene expression level in SALK_018161 (#2, 3) and SALK_115646 plants is presented relative to that of the wild-type plant, which was set at 1.

Supplemental Figure 6

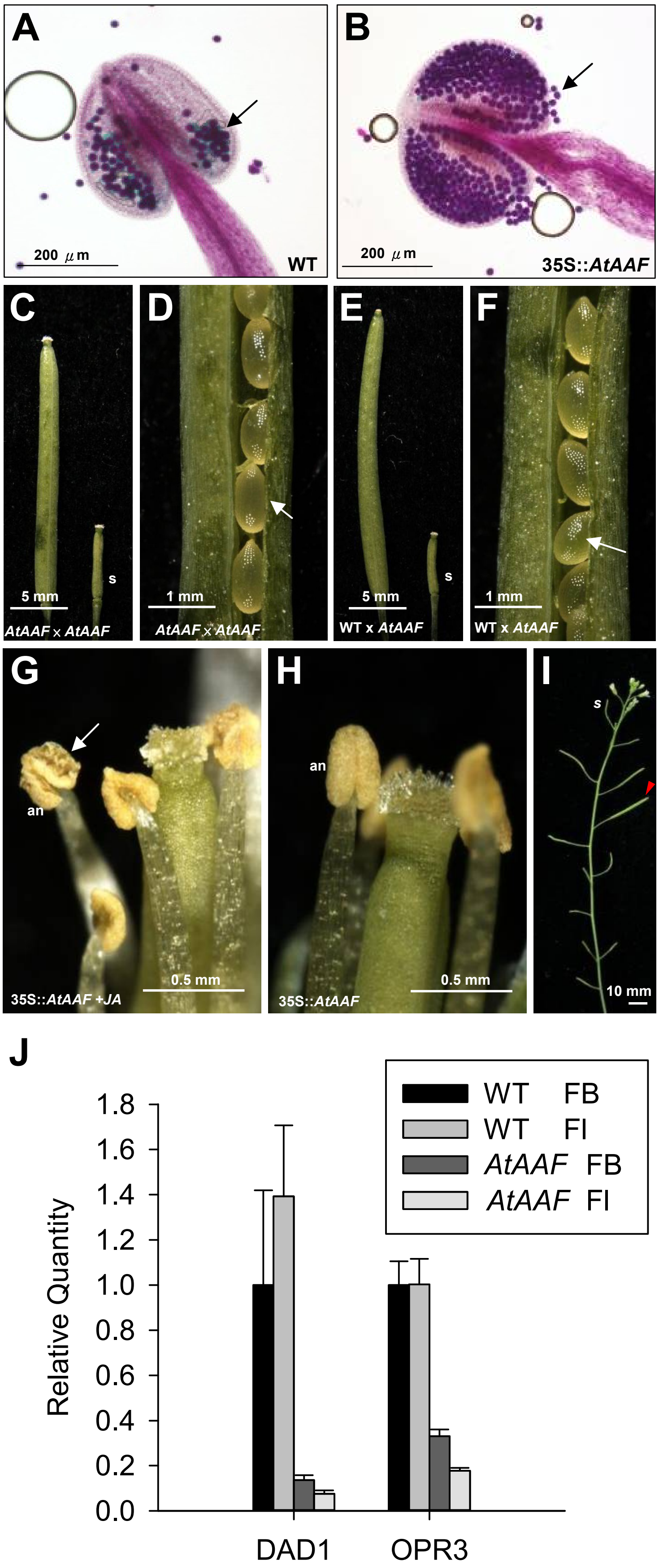

Supplemental Figure 6. Alexander's staining of pollen, phenotypic analysis following JA treatment and the expression of genes involved in JA biosynthesis in 35S::*AtAAF* flowers.

A to B, Pollen grains with normal viability (arrow, stained dark purplish red) were observed in the wild-type dehiscent (A) and 35S::*AtAAF* indehiscent anthers (B) by using Alexander's staining. Bar = 200 μm.

C, The manually self-pollinated 35S::*AtAAF* flowers developed well-elongated siliques (left), whereas short siliques (s) developed without manual pollination. Bar = 5 mm.

D, Normal embryos (arrow) were developed in 35S::*AtAAF* siliques after manual self-pollination (35S::*AtAAF* x 35S::*AtAAF*). Bar = 1 mm.

E, Well-elongated silique (left) was developed in 35S::*AtAAF* flower after manually pollinating with wild-type pollen. Short silique (s) was developed without manual pollination. Bar = 5 mm.

F, Normal embryos (arrow) were developed in 35S::*AtAAF* siliques after manual pollination with wild-type (WT x 35S::*AtAAF*) pollen grains. Bar = 1 mm.

G, The anthers (an) were dehiscent and the pollen (arrow) were released in a 35S::*AtAAF* flower after JA treatment. Bar = 0.5 mm.

H, The anthers (an) were indehiscent in a 35S::*AtAAF* flower without JA treatment. Bar = 0.5 mm.

I, A JA-treated 35S::*AtAAF* flower developed an elongated silique (arrow), whereas short siliques (s) developed without JA treatment. Bar = 10 mm.

J, Detection of the *DAD1* and *OPR3* expression in floral buds (FB) and mature flowers (Fl) of wild-type (WT) and 35S::*AtAAF* (*AtAAF*) plants.

Supplemental Figure 7

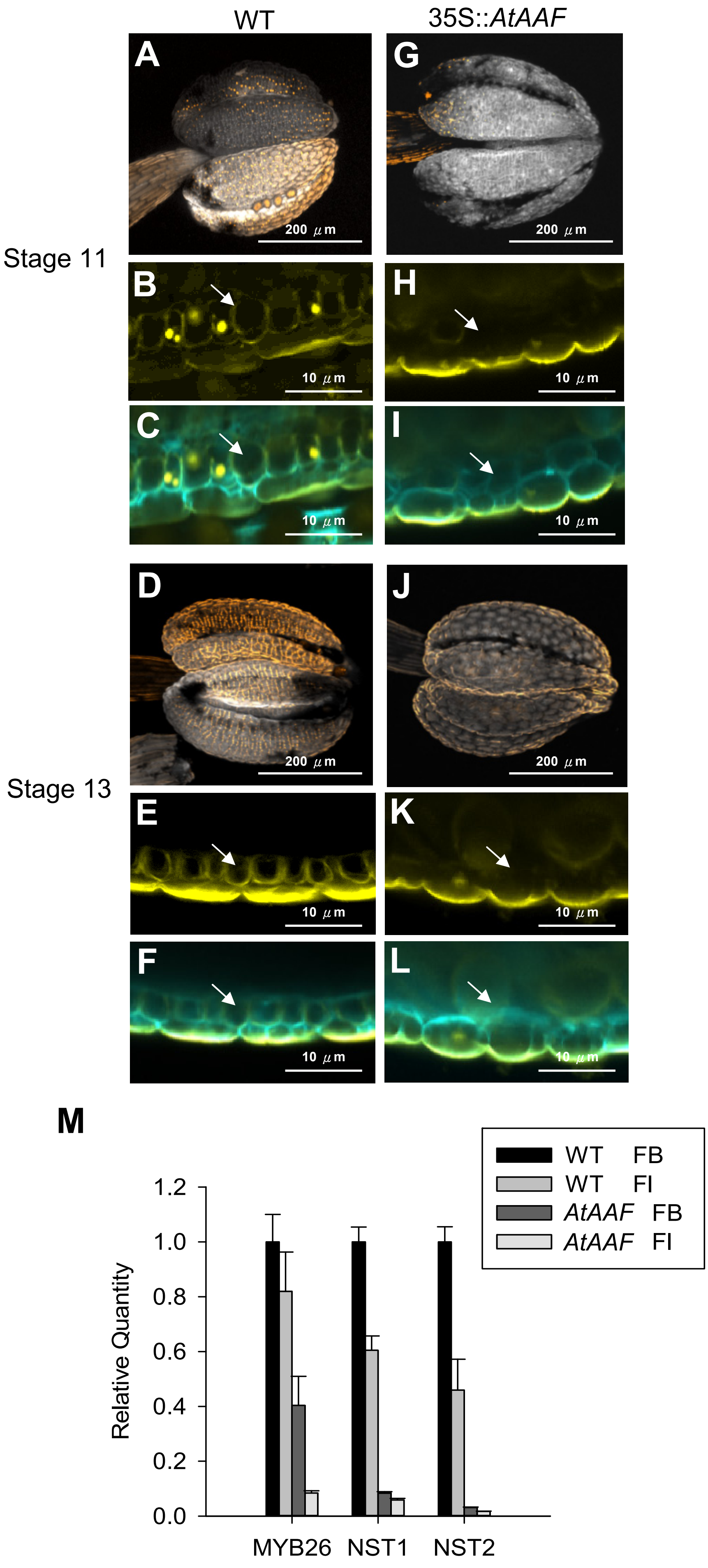

Supplemental Figure 7. Lignin staining of the anther and gene expression in 35S::*AtAAF* flowers.
A to L, Anthers at stage 11 (A to C) and 13 (D to F) of wild-type (WT) and stage 11 (G to I) and 13 (J to L) of 35S::*AtAAF* flowers were stained with auramine O (B, E, H, K) and calcofluor white (C, F, I, L) and observed by confocal microscopy (370 nm excitation/420 emission and 488 nm excitation/510–560 nm emission). Secondary thickening is visible in the endothecium (arrowed) of the anthers of wild-type flowers (B, C, E, F) and is absent in 35S::*AtAAF* flowers (H, I, K, L). Bar = 200 μm in (A, D, G, J); Bar = 10 μm in (B, C, E, F, H, I, K, L).
M, Detection of *MYB26*, *NST1* and *NST2* expression in floral buds (FB) and mature flowers (Fl) of wild-type (WT) and 35S::*AtAAF* (*AtAAF*) plants.

```
        *      20      *      40      *      60      *      80      *     100      *     120      *     140      *     160
proTET9 : gtgtcatcatgcgttatgcttgtcggctttgaccataacgcagagatatagaactagcttttacttaacttttagatttattatttgatctagagttaagtggagatatatagtgtttttgttagattattggtggatgtgagagtttgtctttagtttcaa : 162
          GTGTCATCATGCGTTATGCTTGTCGGCTTTGACCATAACGCAGAGATATAGAACTAGCTTTTACTTAACTTTTAGATTTATTATTTGATCTAGAGTTAAGTGGAGATATATAGTGTTTTTGTTAGATTATTGGTGGATGTGAGAGTTTGTCTTTAGTTTCAA

        *     180      *     200      *     220      *     240      *     260      *     280      *     300      *     320
proTET9 : gttgagaatataaggcaagaggagactctgaggcaatcagaggttttgattggcaaaatatccaaaaggcccaaaccaagtcgaagcccatctcgtacaaaaaaagaaagagatctgtaagaaaaaatattctttgatattcttacaaaaataagtgtaaaa : 324
          GTTGAGAATATAAGGCAAGAGGAGACTCTGAGGCAATCAGAGGTTTTGATTGGCAAAATATCCAAAAGGCCCAAACCAAGTCGAAGCCCATCTCGTACAAAAAAAGAAAGAGATCTGTAAGAAAAAATATTCTTTGATATTCTTACAAAAATAAGTGTAAAA

        *     340      *     360      *     380      *     400      *     420      *     440      *     460      *     480
proTET9 : cttttattagtcaaaatctttaaaaactctcatcactcctacgaaagcgcgtgagagttatgagacattccttaatagcattactcacaagtcacaagttcaaaacgtctgactgaaacagaaacaagcctttgttgaagtcttgaagaagagacattagta : 486
          CTTTTATTAGTCAAAATCTTTAAAAACTCTCATCACTCCTACGAAAGCGCGTGAGAGTTATGAGACATTCCTTAATAGCATTACTCACAAGTCACAAGTTCAAAACGTCTGACTGAAACAGAAACAAGCCTTTGTTGAAGTCTTGAAGAAGAGACATTAGTA

        *     500      *     520      *     540      *     560      *     580      *     600      *     620      *     640
proTET9 : ctcgtcgtatagccataaaaggtaatatacgaaatttcttcgctaatctcttcaccttcctctacgcgtttcactttcactttataaatccaaatctcccttcgaaaacataatcacacaaatcccttttttggtttctccaaatcttcaaatcttcttcaa : 648
          CTCGTCGTATAGCCATAAAAGGTAATATACGAAATTTCTTCGCTAATCTCTTCACCTTCCTCTACGCGTTTCACTTTCACTTTATAAATCCAAATCTCCCTTCGAAAACATAATCACACAAATCCCTTTTTTGGTTTCTCCAAATCTTCAAATCTTCTTCAA

        *     660      *     680      *     700      *     720      *     740      *     760      *     780      *     800      *
proTET9 : tcatcaccATGGTACGTTTTAGTAACAGTCTTGTAGGAATACTCAACTTCTTCGTCTTCCTTCTCTCGGTTCCCATACTCTCAACCGGAATCTGGCTCAGCCTTAAAGCCACGACGCAATGCGAGAGATTCCTCGACAAACCCATGATCGCTCTCGGTGTTT : 810
          TCATCACCATGGTACGTTTTAGTAACAGTCTTGTAGGAATACTCAACTTCTTCGTCTTCCTTCTCTCGGTTCCCATACTCTCAACCGGAATCTGGCTCAGCCTTAAAGCCACGACGCAATGCGAGAGATTCCTCGACAAACCCATGATCGCTCTCGGTGTTT

             820      *     840      *     860      *     880      *     900      *     920      *     940      *     960      *
proTET9 : TCCTCATGATAATCGCAATCGCTGGAGTCGTTGGATCTTGTTGCAGAGTGACGTGGCTTCTCTGGTCCTATCTCTTTGTGATGTTCTTCTTAATCCTCATCGTCCTCTGTTTCACCATCTTTGCCTTCGTTGTCACTAGTAAAGGCTCCGGCGAAACTATCC : 972
          TCCTCATGATAATCGCAATCGCTGGAGTCGTTGGATCTTGTTGCAGAGTGACGTGGCTTCTCTGGTCCTATCTCTTTGTGATGTTCTTCTTAATCCTCATCGTCCTCTGTTTCACCATCTTTGCCTTCGTTGTCACTAGTAAAGGCTCCGGCGAAACTATCC

             980      *    1000      *    1020      *    1040      *    1060      *    1080      *    1100      *    1120      *
proTET9 : AAGGAAAAGCTTATAAGGAGTATAGGCTCGAGGCTTACTCTGATTGGTTGCAGAGGCGTGTGAACAACGCTAAGCATTGGAACAGCATTAGAAGCTGTCTTTATGAGAGCAAGTTCTGTTATAACTTGGAGTTAGTCACTGCTAATCACACTGTTTCTGATT : 1134
          AAGGAAAAGCTTATAAGGAGTATAGGCTCGAGGCTTACTCTGATTGGTTGCAGAGGCGTGTGAACAACGCTAAGCATTGGAACAGCATTAGAAGCTGTCTTTATGAGAGCAAGTTCTGTTATAACTTGGAGTTAGTCACTGCTAATCACACTGTTTCTGATT
```

Supplemental Figure 8. Genomic sequence of *AtAAF* promoter region. Two yellow boxes represent CArG boxes consensus sequence (CC(A/T)$_6$GG) in *AtAAF* promoter region. Blue box represent the start codon ATG for *AtAAF*.

Supplemental Figure 9

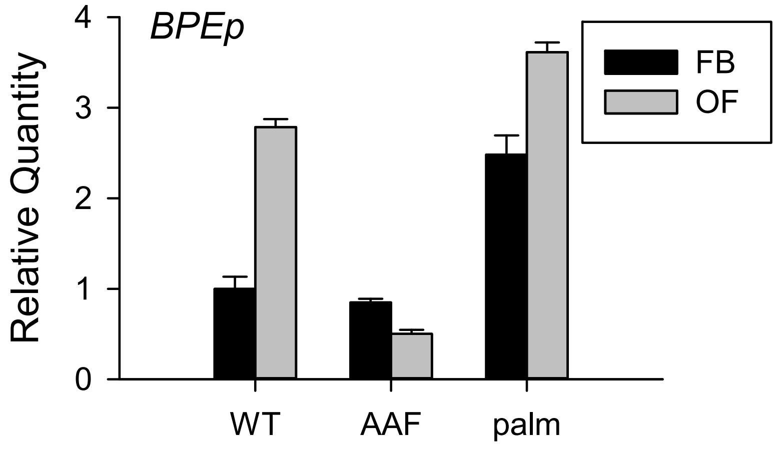

Supplemental Figure 9. Detection of *BPEp* expression in flower buds (FB) and open flower (OF) of wild-type (WT), 35S::*AtAAF* (*AAF)* and 35S::*AtAAF*^{palm} (palm) plants. In wild-type plants, the *BPEp* expression was clearly much higher in open flower (late developmental stage) than in flower buds (early developmental stage). In contrast, *BPEp* expression was low in both open and flower buds of 35S::*AtAAF* plants. *BPEp* expression was however high in both open and flower buds of 35S::*AtAAF*^{palm} plants. This result indicated that *AtAAF* could suppress *BPEp* expression and promote cell expansion during late petal development in 35S::*AtAAF* Arabidopsis.
