# Supplementary material for: A tetraspanin gene regulating auxin response and affecting orchid perianth size and various plant developmental processes
Source: Plant Direct. 2019 Aug 5;3(8):e00157. doi: 10.1002/pld3.157 (PMC6680136; doi:10.1002/pld3.157)
Supplement: Supplementary file 2 [file PLD3-3-e00157-s001.pdf]

Supplemental Table 1. Oligo nucleotide sequence of primers used in gene cloning and PCR analysis.

Gene name	Primer name	Primer sequence	Restriction site	Use
AtAAF At4G30430	pMSIF-1	5’-CTGCAGGGTGGTAAAAAACGTGGAG-3’	PstI	promoter cloning
	pMSIF-2	5’-GTCGACGGTGATGATTGAAGAAGATTTGA-3’	SalI	promoter cloning
	MSIF-3	5’-TCTAGAATCACACAAATCCCTTTTTTGGTT-3’	XbaI	cDNA cloning
	MSIF-4	5’-GGTACCAAACACAAATCAACAGAAGAAATCC-3’	KpnI	cDNA cloning
	MSIF-17	5’-GATCTTCTTCCAGAGTGACGTGGCTTC-3’		C65/66S mutation
	MSIF-18	5’-GAAGCCACGTCACTCTGGAAGAAGATC-3’		C65/66S mutation
	MSIF-19	5’-CTTTTGAGTCTGGTTGCTGCAAGCC-3’		Mega PCR
	MSIF-20	5’-GGCTTGCAGCAACCAGACTCAAAAG-3’		Mega PCR
	MSIF-22	5’-GTCTACGCTATGGGATCTTCCGCTTTC-3’		C252/253S mutation
	MSIF-21	5’-GAAAGCGGAAGATCCCATAGCGTAGAC-3’		C252/253S mutation
	MSIF-fusion	5’-GGTACCAGAATTGTTGAAACCATTGGAACG-3’	KpnI	cDNA cloning
	RT-AtAAF-F	5’-GGAGTTAGTCACTGCTAATCACAC-3’		Real-time PCR
	RT-AtAAF-R	5’-ACGACAACGAGGAGTACAAGG-3’		Real-time PCR
OnAAF	OnAAF-1	5’-AGTCTAGACATGGTGCGGATCAGC-3’	XbaI	cDNA cloning
	OnAAF-2	5’-ACGGTACCTATGGGTTCCCTCCCT-3’	KpnI	cDNA cloning
	RT-OnAAF-F	5’-ATGGCTTCTATGGCTGTA-3’		Real-time PCR
	RT-OnAAF-R	5’-TCTGGTTCTTCTCACTCA-3’		Real-time PCR
PaAAF	PaAAF-1	5’-AGTCTAGAATGGTTCGGTTCAGCAATAACC-3’	XbaI	cDNA cloning
	PaAAF-2	5’-ACGGTACCTAAGGGTTCCCTCCCTT-3’	KpnI	cDNA cloning
	RT-PaAAF-F	5’-TTGGTCTAAGATTCGGAGTTG-3’		Real-time PCR
	RT-PaAAF-R	5’-GAAGAGGAGTAAGATTATCGTTGA-3’		Real-time PCR
OAGL6-2	RT-OAGL6-2-F	5’-GAGCACTTCAAGGTTCCAACTG-3’		Real-time PCR
	RT-OAGL6-2-R	5’-TTGATCACATTCTCTTAAACAGCCCA -3’		Real-time PCR
PeMADS9	RT-PeM9-F	5'-CTTCCTGCAATGTAATTTCAGCTGT-3'		Real-time PCR
	RT-PeM9-R	5'-AACCAGTCATAGTGTAAAAGTTGAGAA-3'		Real-time PCR
EDF1 At1G25560	RT-EDF1-F	5’-TCACAAACACAACAAATATGGAATACAG-3’		Real-time PCR
	RT-EDF1-R	5’-GCTTTGGAGTAGTAGAGATGGAGAG-3’		Real-time PCR
EDF2 At1G68840	RT-EDF2-F	5’-CTAAACAACACGCCGAGAAACAC-3’		Real-time PCR
	RT-EDF2-R	5’-GCCGAACCACCACCTGAACC-3’		Real-time PCR
ERF1 At3G23240	RT-ERF1-F	5’-GAGCAGTCCACGCAACAAAC-3’		Real-time PCR
	RT-ERF1-R	5’-TCTCCGAAAGCGACTCTTGAAC-3’		Real-time PCR
SAG12 At5G45890	RT-SAG12-F	5’-TTACAGGTTATGAGGATG-3’		Real-time PCR
	RT-SAG12-R	5’-AGACGAATAGAATTGGAA-3’		Real-time PCR

GFP	RT-GFP-F	5’-GAAGATGGAAGCGTTCAA-3’		Real-time PCR
	RT-GFP-R	5’-AGGTAATGGTTGTCTGGTA-3’		Real-time PCR
DAD1 At2G44810	RT-DAD1-F	5’AGATCGCAAGGCTACTCCAATC3’		Real-time PCR
	RT-DAD1-R	5’CCACGGCGTCTCCTCCAC3’		Real-time PCR
OPR3 At2g06050	RT-OPR3-F	5’-TGGACGCAACTGATTCTGACC-3’		Real-time PCR
	RT-OPR3-R	5’-TGGTAGCGAGGTTGTGTAACG-3’		Real-time PCR
MYB26 At3g13890	RT-MYB26-F	5’-TCCATCCTCTTCTTCTTCATCTACC-3’		Real-time PCR
	RT-MYB26-R	5’-TCTCAGCACTTGACGCATATACC-3’		Real-time PCR
UBQ10 At4G05320	RT-UBQ10-F	5'-CTCAGGCTCCGTGGTGGTATG-3'		Real-time PCR
	RT-UBQ10-R	5'-GTGATAGTTTTCCCAGTCAACGTC-3'		Real-time PCR
NST1 At2g46770	RT-NST1-F	5’-GCTTAACGGACCCACATCATATTC-3’		Real-time PCR
	RT-NST1-R	5’-TACGGAGATCGGACGGAAGG-3’		Real-time PCR
NST2 At3g61910	RT-NST2-F	5’-CGCCGTCGTTCAATGAGGAG-3’		Real-time PCR
	RT-NST2-R	5’-TCGTGATGGTGGTGTTGTTATGG-3’		Real-time PCR
PaACTIN4	RT-PACT4-F	5’-GGCTAACAGAGAGAAGATGACC-3’		Real-time PCR
	RT-PACT4-R	5’-AATAGACCCTCCAATCCAGAC-3’		Real-time PCR
OnTubulin	RT-OTUB-F	5’-AGGGCTTTCTGGTTTTCAATGCTGT-3’		Real-time PCR
	RT-OTUB-R	5’-CGGCGACGGCAGTGTTGTT-3’		Real-time PCR
BIGPETAL At1g59640	RT-BPEp-F	5’-TCATCAACTCAATCTTCTCCAT-3’		Real-time PCR
	RT-BPEp-R	5’-AATGACCGAACCGCATAA-3’		Real-time PCR
